# A Concise Synthesis of Pyrrole-Based Drug Candidates from α-Hydroxyketones, 3-Oxobutanenitrile, and Anilines

**DOI:** 10.3390/molecules28031265

**Published:** 2023-01-28

**Authors:** Mengxin Xia, Mardi Santoso, Ziad Moussa, Zaher M. A. Judeh

**Affiliations:** 1 School of Chemistry, Chemical Engineering and Biotechnology, Nanyang Technological University, 62 Nanyang Drive, N1.2-B1-14, Singapore 637459, Singapore; 2 Department of Chemistry, Faculty of Science, Institut Teknologi Sepuluh Nopember, Sukolilo, Surabaya 60111, Indonesia; 3 Department of Chemistry, College of Science, United Arab Emirates University, Al-Ain P.O. Box 15551, United Arab Emirates

**Keywords:** pyrroles, three-component reactions, BM212

## Abstract

A simple and concise three-component synthesis of a key pyrrole framework was developed from the reaction between α-hydroxyketones, oxoacetonitriles, and anilines. The synthesis was used to obtain several pyrrole-based drug candidates, including COX-2 selective NSAID, antituberculosis lead candidates BM212, BM521, and BM533, as well as several analogues. This route has potential to obtain diverse libraries of these pyrrole candidates in a concise manner to develop optimum lead compounds.

## 1. Introduction

**Figure 1:**
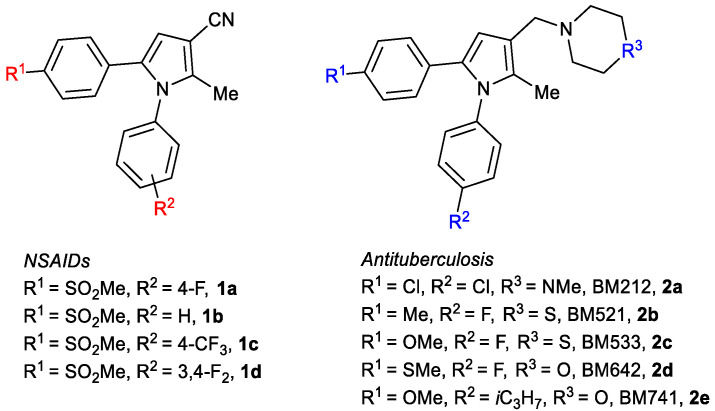
Examples of pyrrole-based drug lead compounds and drug candidates.

The practical synthesis of drug lead compounds in a concise manner is crucial for biological evaluation and the development of structure activity relationship (SAR) studies for further optimization. This becomes even more crucial when developing an industrial synthetic route for a specific drug. Several pyrrole-based drugs have already been introduced in the market with great success [1,2,3]. Additionally, many pyrrole-based lead compounds demonstrated positive bioactivities, such as anti-inflammatory [4,5], anti-bacterial [6], and anti-cancer [7] activities. For example, pyrrole-based compounds **1a**–**d** have demonstrated selective cyclooxygenases (COX-2) inhibition with nonsteroidal anti-inflammatory activity [4,5,6,7,8], while pyrrole-based compounds **2a**–**e** have demonstrated antituberculosis activities [9,10] (Figure 1).

**Scheme 1:**
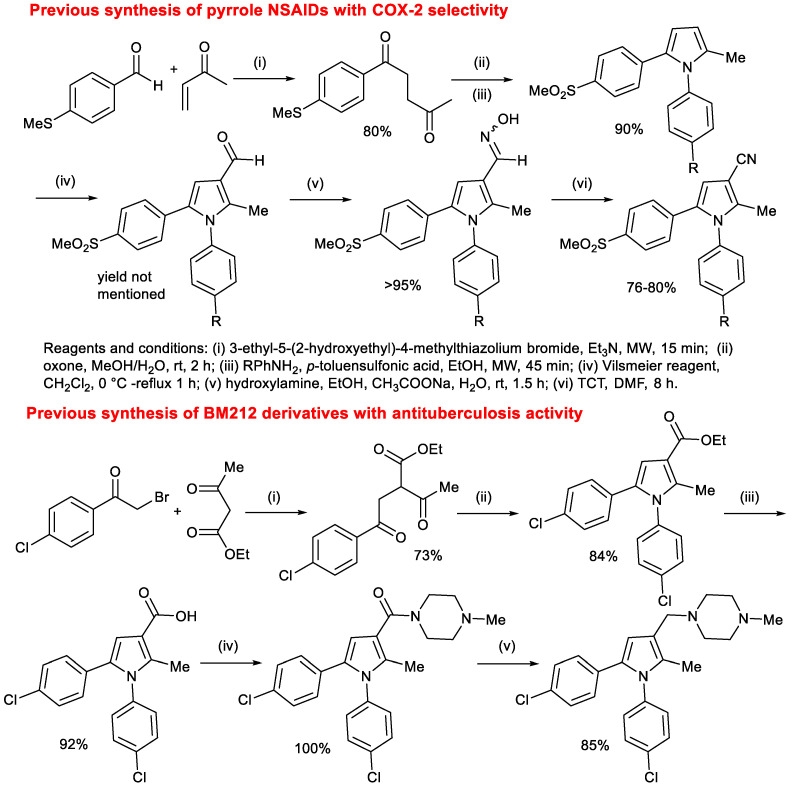
Examples of previous syntheses of pyrrole-based bioactive compounds [4,12].

Several procedures were reported to obtain pyrrole COX-2 selective NSAIDs. However, the reported syntheses are lengthy and gave low overall yields, making SAR studies difficult and synthesis at large scale less practical. Typical syntheses of pyrroles possessing COX-2 [4] and antituberculosis inhibitory activities [11] are shown in Scheme 1. Therefore, considering the problems associated with the current synthetic routes and the potential of pyrrole-based drugs, a practical and concise synthetic route is highly advantageous.

Previously, we reported the selective synthesis of *N*-substituted 2,3,5-functionalized 3-cyanopyrroles *via* a one-step, three-component reaction between α-hydroxyketones, oxoacetonitriles, and primary amines [13]. The mild reaction conditions (AcOH as a catalyst, EtOH, 70 °C, 3 h), applicability on a large scale, and high atom efficiency (water is the only molecule lost during the reaction) warranted the application of this synthesis to important pyrrole-based lead drug candidates [13]. In this work, we report the synthesis of a key pyrrole framework and develop it further for the synthesis of several pyrrole lead drug candidates, including COX-2 selective inhibitor, antituberculosis lead candidates BM212 **2a**, BM521 **2b**, and BM533 **2c**, and several analogues.

## 2. Results and Discussions

**Scheme 2:**
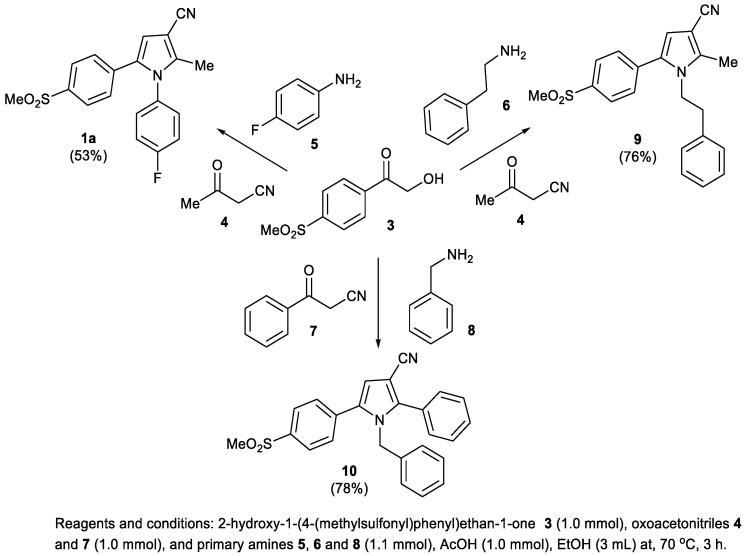
Synthesis of 5-(4-methylsulfonyl)phenyl-substituted pyrroles **1a**, **9**, and **10**.

The AcOH-catalyzed, one-pot, three-component reaction between 2-hydroxy-1-(4-(methylsulfonyl)phenyl)ethan-1-one **3**, 3-oxobutanenitrile **4**, and 4-fluoroaniline **5** gave the anticipated COX-2 selective NSAID pyrrole **9** in 53%. This one-step reaction is very powerful, since a library of analogues of pyrrole **1a** can be prepared using the same procedure by changing the nitrile and amine substrates. For example, the reaction between 2-hydroxy-1-(4-(methylsulfonyl)phenyl)ethan-1-one **3**, 3-oxobutanenitrile **4**, and 2-phenylethylamine **6** gave pyrroles **10** in 76% yield, while the same reaction using 3-oxo-3-phenylpropanenitrile **7** and benzylamine **8** gave pyrrole **11** in 78% yield. (Scheme 2) Additionally, the cyano group can easily be transformed into aldehyde, alcohol, amide, and acid functional groups that act as handles for further structural modifications [8].

**Scheme 3:**
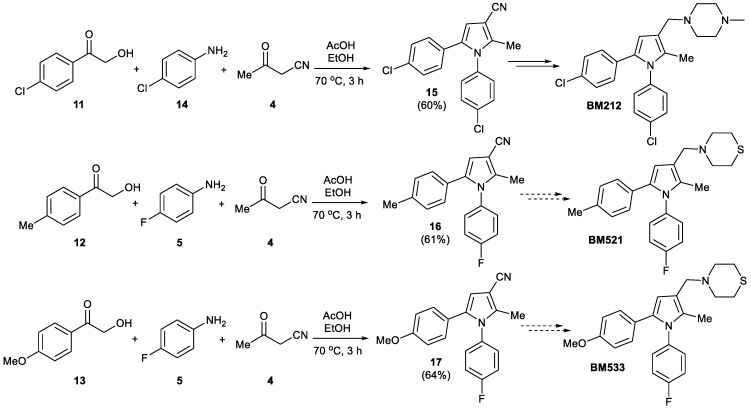
Synthesis of pyrroles **15**–**17**.

We adapted the same route to synthesize BM212 **2a**, BM521 **2b**, and BM533 **2c** framework. Thus, the reaction between phenacyl alcohols **11**–**13**, 3-oxobutanenitrile **4**, and anilines **5** and **14** gave pyrroles **15**–**17** in 60–64% yield. Pyrroles **15**–**17,** which are analogues of pyrrole **1a,** are precursors for BM212, BM521, and BM533, respectively (Scheme 3).

**Scheme 4:**
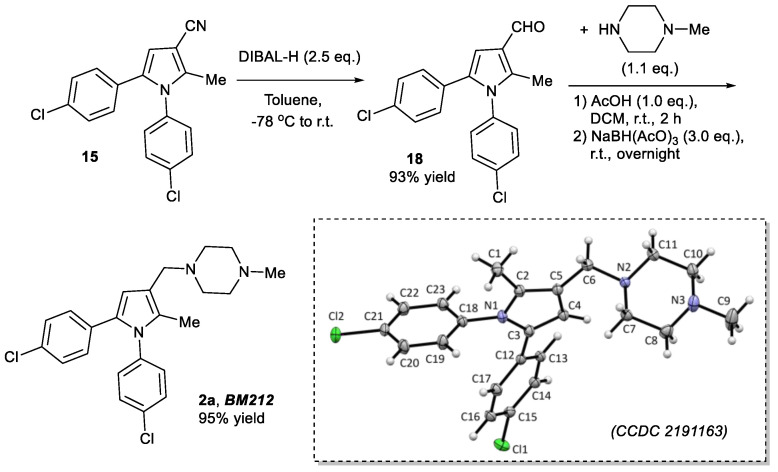
Synthesis of BM212 **2a** from pyrrole product **15**, and X-ray single crystal structure of BM212 **2a** (CCDC 2191163) (displacement ellipsoids are drawn at the 50% probability level).

To demonstrate the feasibility of converting compounds **15**, **16**, and **17** to BM212 **2a**, BM521 **2b**, and BM533 **2c**, respectively, and hence the power of the three-component reaction, we demonstrated the synthesis of BM212 **2a** from pyrrole **15**. Hence, the cyano group of pyrrole **15** was reduced using diisobutylaluminium hydride (DIBAL-H) to give carbaldehyde **18** in 93% yield (Scheme 4) [14]. When carbaldehyde **18** was treated with 1-methylpiperazine in the presence of AcOH in DCM for 2 h, followed by the addition of NaBH(AcO)_3_ and stirring overnight, BM212 **2a** was obtained in 95% yield after silica gel column chromatographic purification [12]. BM521 **2b** and BM533 **2c** can be obtained in a similar fashion from pyrroles **16** and **17,** respectively. α-Hydroxyketones **3** and **11**–**13** were synthesized from the corresponding substituted phenacyl bromide using sodium formate (General Procedure 1, experimental section and [13]).

## 3. Materials and Methods

### 3.1. Materials and Methods

All chemicals and AR grade solvents were obtained from Sigma-Aldrich (Saint Louis, MO, USA), Merck (Lebanon, NJ, USA), or Alfa Aesar (Tewksbury, MA, USA) and were used as received without further purification. IR spectra were recorded using a Bruker MPA FT-IR machine (Karlsruhe, Germany). ^1^H NMR spectra were recorded at 400 MHz Bruker Avance III 400 (BBFO 400). ^13^C NMR spectra were recorded at 101 MHz Bruker Avance III 400 (BBFO 400). HRMS were measured using a hybrid Quadrupole Time-of-Flight (Q-TOF) on a Qstar XL MS/MS system (Milford, CT, USA). Single-crystal X-ray crystallographic analysis was done using Bruker D8 Quest (Karlsruhe, Germany). Analytical TLC was performed using Merck 60 F_254_ precoated silica gel plates (0.2 mm thickness) (Oakville, ON, Canada). The plates were visualized under UV (254 nm) or stained in ceric ammonium sulfate solution with heating to detect the reaction spots. Flash chromatography was performed using Merck silica gel 60 (230–400 mesh) (Oakville, ON, Canada). Copies of the ^1^H NMR, ^13^C NMR, and single-crystal X-ray data of pyrrole **20** can be found in the Supplementary Materials.

### 3.2. General Procedure 1: Preparation of Substituted Phenacyl Alcohols **3** and **11**–**13**

**Scheme 5:**
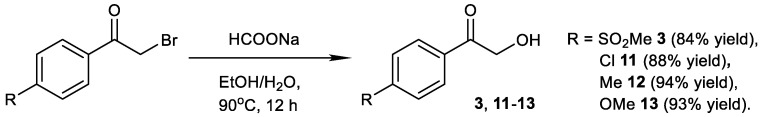
Preparation of phenacyl alcohols **3** and **11**-**13**.

A solution of the phenacyl bromide (20 mmol) and sodium formate (16 mmol) in an ethanol/water mixture (30 mL, EtOH: H_2_O = 9:1) was stirred at 90 °C for 12 h (Scheme 5). Once the reaction was completed (TLC), the mixture was allowed to cool to room temperature and ethanol was removed under vacuum. Water (30 mL) was added to the residue, and the resulting mixture was extracted with ethyl acetate (3 × 30 mL). The combined organic layers were dried over Mg_2_SO_4_ and the solvent was evaporated using under vacuum. The residue was then purified using column chromatography using EtOAc/Hexane as the eluent (2:3 for **3**; 1:4 for **11**–**13**).

### 3.3. General Procedure 2: Synthesis of N-Substituted 1,2,3,5-Substituted Pyrroles **1a**, **9**–**10** and **15**–**17**

AcOH (1.0 eq.) was added dropwise to a stirred solution of substituted phenacyl alcohols **3** and **11**–**13** (1.0 eq.), oxoacetonitriles **4** and **7** (1.0 eq.), and primary amine **5**, **6**, **8,** and **14** (1.1 eq.) in EtOH (3 mL) at room temperature. The resulting mixture was heated at 70 °C for 3 h (TLC). The reaction mixture was then evaporated to dryness under vacuum to give the crude product as a foam. The foam was purified using silica gel column chromatography with 5–35% EtOAc/Hexane as eluent t yield the pure products. All reactions were conducted using 1.0 mmol of the substrates **3** and **11**–**13**. This general procedure was used to prepare *N*-substituted 2,3,5-functionalized pyrroles **1a**, **9**–**10** and **15**–**17**.

1-(4-Fluorophenyl)-2-methyl-5-(4-(methylsulfonyl)phenyl)-1*H*-pyrrole-3-carbonitrile **1a**


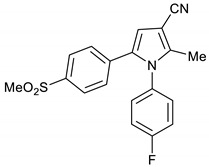


Obtained by General Procedure 2 using 2-hydroxy-1-(4-(methylsulfonyl)phenyl)ethan-1-one **3** (214 mg, 1.0 mmol), 3-oxobutanenitrile **4** (83 mg, 1.0 mmol), 4-fluoro aniline **5** (104 µL, 1.1 mmol), and AcOH (57 µL, 1.0 mmol). The reaction gave the desired **1a** (334 mg, 53%) as a hygroscopic pale-yellow solid. IR (KBr film) *ѵ*_max_ = 2221, 1513, 1402, 1306, 1152 cm^−1^. ^1^H NMR (400 MHz, CDCl_3_) δ 7.78 (d, *J* = 8.6 Hz, 2H), 7.21 (d, *J* = 8.6 Hz, 2H), 7.17 (dd, *J* = 6.3, 2.0 Hz, 4H), 6.71 (s, 1H), 3.04 (s, 3H), 2.31 (s, 3H). ^13^C NMR (101 MHz, CDCl_3_) δ 163.8, 161.3, 143.7, 141.5, 138.9, 136.5, 133.1, 129.8, 129.7, 128.4, 127.6, 117.2, 116.9, 116.2, 112.3, 93.7, 44.4, 12.4. DEPT135 ^13^C NMR (101 MHz, CDCl_3_) δ 129.8, 129.7, 128.4, 127.6, 120.0, 117.2, 116.9, 112.3, 44.4, 12.4. HRMS (ESI-TOF) m/z: [M+H]^+^ calculated for C_19_H_16_N_2_O_2_SF^+^ 355.0917; found 355.0907.

2-Methyl-5-(4-(methylsulfonyl)phenyl)-1-phenethyl-1*H*-pyrrole-3-carbonitrile **9**


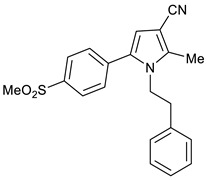


Obtained by General Procedure 2 using 2-hydroxy-1-(4-(methylsulfonyl)phenyl)ethan-1-one **3** (214 mg, 1.0 mmol), 3-oxobutanenitrile **4** (83 mg, 1.0 mmol), 2-phenylethylamine **6** (138 µL, 1.1 mmol), and AcOH (57 µL, 1.0 mmol). The reaction gave the desired **9** (277 mg, 76%) as a hygroscopic pale-yellow solid. IR (KBr film) *ѵ*_max_ = 2929, 2218, 1598, 1426, 1311, 1150, 1092 cm^−1^. ^1^H NMR (400 MHz, CDCl_3_) δ 7.96 (d, *J* = 7.8 Hz, 2H), 7.39 (d, *J* = 7.8 Hz, 2H), 7.21 (d, *J* = 6.4 Hz, 3H), 6.78 (d, *J* = 6.7 Hz, 2H), 6.39 (s, 1H), 4.18 (t, *J* = 6.7 Hz, 2H), 3.12 (s, 3H), 2.74 (t, *J* = 6.6 Hz, 2H), 2.31 (s, 3H). ^13^C NMR (101 MHz, CDCl_3_) δ 139.9, 139.7, 137.5, 136.6, 132.7, 129.7, 128.8, 128.6, 127.8, 127.2, 116.7, 111.9, 92.5, 46.4, 44.5, 36.8, 11.7. DEPT135 ^13^C NMR (101 MHz, CDCl_3_) δ 129.7, 128.8, 128.6, 127.8, 127.2, 111.9, 46.4, 44.5, 36.8, 11.7. HRMS (ESI-TOF) m/z: [M+H]^+^ calculated for C_21_H_21_N_2_O_2_S^+^ 365.1324; found 365.1319.

1-Benzyl-5-(4-(methylsulfonyl)phenyl)-2-phenyl-1*H*-pyrrole-3-carbonitrile **10**


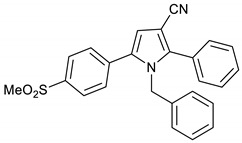


Obtained by General Procedure 2 using 2-hydroxy-1-(4-(methylsulfonyl)phenyl)ethan-1-one **3** (214 mg, 1.0 mmol), benzoylacetonitrile **7** (145 mg, 1.0 mmol), benzylamine **8** (120 µL, 1.1 mmol), and AcOH (57 µL, 1.0 mmol). The reaction gave the desired **10** (322 mg, 78%) as a hygroscopic pale-yellow solid. IR (KBr film) *ѵ*_max_ = 2222, 1602, 1403, 1313, 1151 cm^−1^. ^1^H NMR (400 MHz, CDCl_3_) δ 7.95–7.89 (m, 2H), 7.53–7.49 (m, 2H), 7.44 (s, 5H), 7.19 (dd, *J* = 4.2, 2.3 Hz, 3H), 6.71 (s, 1H), 6.64 (dd, *J* = 6.6, 2.9 Hz, 2H), 5.20 (s, 2H), 3.08 (s, 3H). ^13^C NMR (101 MHz, CDCl_3_) δ 144.3, 140.0, 137.1, 136.9, 134.4, 129.8, 129.7, 129.6, 129.1, 129.0, 128.8, 127.8, 127.7, 125.7, 116.5, 113.6, 94.1, 49.5, 44.5. DEPT135 ^13^C NMR (101 MHz, CDCl_3_) δ 129.8, 129.7, 129.6, 129.0, 128.8, 127.8, 127.8, 125.7, 113.6, 49.5, 44.5. HRMS (ESI-TOF) m/z: [M+H]^+^ calculated for C_25_H_18_N_2_O_3_F^+^ 413.1301; found 413.1281.

1,5-Bis(4-chlorophenyl)-2-methyl-1*H*-pyrrole-3-carbonitrile **15**


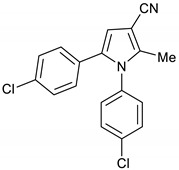


Obtained by General Procedure 2 using 1-(4-chlorophenyl)-2-hydroxyethan-1-one **11** (170 mg, 1.0 mmol), 3-oxobutanenitrile **4** (83 mg, 1.0 mmol), 4-chloro aniline **14** (141 mg, 1.1 mmol), and AcOH (57 µL, 1.0 mmol). The reaction gave the desired **15** (209 mg, 64%) as a hygroscopic pale-yellow solid. IR (KBr film) *ѵ*_max_ = 2218, 1651, 1496, 1400, 1094 cm^−1^. ^1^H NMR (400 MHz, CDCl_3_) δ 7.42 (d, *J* = 8.6 Hz, 2H), 7.20 (d, *J* = 8.5 Hz, 2H), 7.08 (d, *J* = 8.6 Hz, 2H), 6.96 (d, *J* = 8.5 Hz, 2H), 6.55 (s, 1H), 2.29 (s, 3H). ^13^C NMR (101 MHz, CDCl_3_) δ 140.1, 135.8, 135.0, 134.0, 133.5, 129.9, 129.6, 129.5, 129.3, 128.7, 116.6, 110.7, 93.2, 12.4. DEPT135 ^13^C NMR (101 MHz, CDCl_3_) δ 129.9, 129.5, 129.3, 128.7, 110.7, 12.4. HRMS (ESI-TOF) m/z: [M+H]^+^ calculated for C_18_H_13_N_2_Cl_2_^+^ 327.0456; found 327.0451.

1-(4-Fluorophenyl)-2-methyl-5-(*p*-tolyl)-1*H*-pyrrole-3-carbonitrile **16**


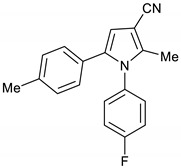


Obtained by General Procedure 2 using 2-hydroxy-1-(*p*-tolyl)ethan-1-one **12** (150 mg, 1.0 mmol), 3-oxobutanenitrile **4** (83 mg, 1.0 mmol), 4-fluoro aniline **5** (104 µL, 1.1 mmol), and AcOH (57 µL, 1.0 mmol). The reaction gave the desired **16** (174 mg, 60%) as a hygroscopic pale-yellow solid. IR (KBr film) *ѵ*_max_ = 2223, 1604, 1512, 1403, 1227 cm^−1^. ^1^H NMR (400 MHz, CDCl_3_) δ 7.16–7.08 (m, 4H), 7.02 (d, *J* = 8.0 Hz, 2H), 6.92 (d, *J* = 8.1 Hz, 2H), 6.51 (s, 1H), 2.30 (s, 3H), 2.28 (s, 3H). ^13^C NMR (101 MHz, CDCl_3_) δ 163.5, 161.0, 139.6, 137.3, 135.4, 133.6, 129.9, 129.8, 129.0, 128.3, 127.3, 117.0, 116.6, 116.4, 109.8, 92.6, 21.1, 12.4. DEPT135 ^13^C NMR (101 MHz, CDCl_3_) δ 129.9, 129.8, 129.0, 128.3, 116.6, 116.4, 109.8, 21.1, 12.4. HRMS (ESI-TOF) m/z: [M+Na]^+^ calculated for C_19_H_15_N_2_FNa^+^ 313.1117; found 313.1111.

1-(4-Fluorophenyl)-5-(4-methoxyphenyl)-2-methyl-1*H*-pyrrole-3-carbonitrile **17**


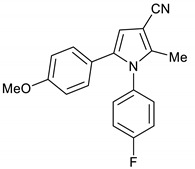


Obtained by General Procedure 2 using 2-hydroxy-1-(4-methoxyphenyl)ethan-1-one **13** (166 mg, 1.0 mmol), 3-oxobutanenitrile **4** (83 mg, 1.0 mmol), 4-fluoro aniline **5** (104 µL, 1.1 mmol), and AcOH (57 µL, 1.0 mmol). The reaction gave the desired **17** (187 mg, 61%) as a hygroscopic pale-yellow solid. IR (KBr film) *ѵ*_max_ = 2218, 1614, 1511, 1403, 1248 cm^−1^. ^1^H NMR (400 MHz, CDCl_3_) δ 7.12 (d, *J* = 6.3 Hz, 4H), 6.96 (d, *J* = 8.8 Hz, 2H), 6.74 (d, *J* = 8.8 Hz, 2H), 6.47 (s, 1H), 3.78 (s, 3H), 2.27 (s, 3H). ^13^C NMR (101 MHz, CDCl_3_) δ 163.4, 161.0, 158.9, 139.3, 135.2, 129.9, 129.9, 129.7, 128.6, 123.8, 116.6, 116.3, 114.1, 113.8, 109.4, 92.5, 55.2, 12.4. DEPT135 ^13^C NMR (101 MHz, CDCl_3_) δ 129.9, 129.9, 129.7, 116.6, 116.4, 113.8, 109.4, 55.2, 12.4. HRMS (ESI-TOF) m/z: [M+Na]^+^ calculated for C_19_H_15_N_2_OFNa^+^ 329.1066; found 329.1069.

1,5-Bis(4-chlorophenyl)-2-methyl-1*H*-pyrrole-3-carbaldehyde **18**


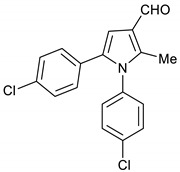


DIBAL-H (1.0 M solution in THF, 2.5 mL, 2.5 mmol) was added slowly to a solution of pyrrole **15** (326 mg, 1.0 mmol) in toluene (10 mL) at −78 °C under stirring. The reaction mixture was allowed to stir at room temperature for 2 h, quenched with saturated K_2_CO_3_ solution (15 mL) and then extracted with ethyl acetate (3 × 15 mL). The organic layers were dried using MgSO_4_ then evaporated under vacuum. This crude product was purified by column chromatography using Hexane/EtOAc (4:1) and gave **18** (307 mg, 93%) as a hygroscopic pale-yellow solid. IR (KBr film) *ѵ*_max_ = 1718, 1559, 1401, 1093 cm^−1^. ^1^H NMR (400 MHz, CDCl_3_) δ 9.99 (s, 1H), 7.43 (d, *J* = 8.4 Hz, 2H), 7.19 (d, *J* = 8.4 Hz, 2H), 7.11 (d, *J* = 8.4 Hz, 2H), 6.98 (d, *J* = 8.3 Hz, 2H), 6.80 (s, 1H), 2.42 (s, 3H). ^13^C NMR (101 MHz, CDCl_3_) δ 185.6, 140.0, 135.7, 134.9, 134.6, 133.2, 130.1, 129.8, 129.5, 129.4, 128.6, 123.0, 109.0, 11.5. DEPT135 ^13^C NMR (101 MHz, CDCl_3_) δ 185.6, 129.8, 129.5, 129.4, 128.6, 109.0, 11.5. HRMS (ESI-TOF) m/z: [M+H]^+^ calculated for C_18_H_14_NOCl_2_^+^ 330.0452; found 330.0457.

1-((1,5-Bis(4-chlorophenyl)-2-methyl-1*H*-pyrrol-3-yl)methyl)-4-methylpiperazine **2a**


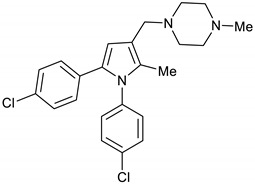


AcOH (57 µL, 1 mmol) and 1-methylpiperazine (122 µL, 1.1 mmol) were added to a stirring solution of pyrrole **18** (309 mg, 0.94 mmol) in DCM (10 mL) at room temperature. The mixture was allowed to stir at room temperature for 2 h before NaBH(AcO)_3_ (636 mg, 3 mmol) was added and the mixture was left to stir overnight. The reaction mixture was then quenched with 1M NaOH solution (15 mL), stirred for 30 min., diluted with water (10 mL) and extracted with DCM (2 X 10 mL). The organic layers were dried over Na_2_SO_4_ and evaporated under vacuum. The crude product was purified by silica gel column chromatography using EtOAc/MeOH (4:1) to give the desired BM212 **2a** (366 mg, 95%) as a hygroscopic pale-yellow solid. IR (KBr film) *ѵ*_max_ = 2935, 2792, 1493, 1167, 1087, 1014 cm^−1^. ^1^H NMR (400 MHz, CDCl_3_) δ 7.36 (d, *J* = 8.6 Hz, 2H), 7.13 (d, *J* = 8.6 Hz, 2H), 7.07 (d, *J* = 8.6 Hz, 2H), 6.96 (d, *J* = 8.6 Hz, 2H), 6.37 (s, 1H), 3.48 (s, 2H), 2.52 (s, 8H), 2.32 (s, 3H), 2.08 (s, 3H). ^13^C NMR (101 MHz, CDCl_3_) δ 137.8, 133.4, 131.9, 131.6, 131.5, 130.2, 129.7, 129.3, 128.8, 128.3, 116.9, 111.7, 55.1, 54.3, 52.7, 46.0, 11.2. DEPT135 ^13^C NMR (101 MHz, CDCl_3_) δ 129.7, 129.3, 128.8, 128.3, 111.7, 55.1, 54.3, 52.7, 46.0, 11.2. HRMS (ESI-TOF) m/z: [M+H]^+^ calculated for C_23_H_26_N_3_Cl_2_^+^ 414.1504; found 414.1486.

## 4. Conclusions

A concise synthetic route for pyrrole-based drug candidates from α-hydroxyketones, 3-oxobutanenitrile, and anilines is presented. The route was demonstrated by the synthesis of several drugs, with high overall yields. This route provides an easy strategy to quickly obtain pyrrole-based drug candidates and has wider applications to the synthesis of analogue compounds.

## Data Availability

Data is published in the Supporting Material in this Journal under Supplementary Materials.

## Supplementary Materials

The following supporting information can be downloaded at: https://www.mdpi.com/article/10.3390/molecules28031265/s1. Copies of the ^1^H NMR, ^13^C NMR, and single-crystal X-ray data of pyrrole **20** are available online.

## Sample Availability

Samples of the compounds are available from the authors.
