# Supplementary material for: A Concise Synthesis of Pyrrole-Based Drug Candidates from α-Hydroxyketones, 3-Oxobutanenitrile, and Anilines
Source: Molecules. 2023 Jan 28;28(3):1265. doi: 10.3390/molecules28031265 (PMC9918993; doi:10.3390/molecules28031265)
Supplement: Supplementary file 1 [file molecules-28-01265-s001.zip › molecules-2138063-supplementary.pdf]

molecules

MDPI

Communication

A Concise Synthesis of Pyrrole-Based Drug Candidates from α-Hydroxyketones, 3-Oxobutanenitrile, and Anilines

Mengxin Xia [1], Mardi Santoso [2,*], Ziad Moussa [3] and Zaher M. A. Judeh [1,*]

[1] School of Chemistry, Chemical Engineering and Biotechnology, Nanyang Technological University, 62 Nanyang Drive, N1.2-B1-14, Singapore 637459, Singapore
[2] Department of Chemistry, Faculty of Science, Institut Teknologi Sepuluh Nopember, Sukolilo, Surabaya 60111, Indonesia
[3] Department of Chemistry, College of Science, United Arab Emirates University, Al-Ain P.O. Box 15551, United Arab Emirates
* Correspondence: tsv09@yahoo.com (M.S.); zaher@ntu.edu.sg (Z.M.A.J.); Tel.: +65-6790-6738 (Z.M.A.J.); Fax: +65-6794-7553 (Z.M.A.J.)

Table of Contents:

	Page
1. ^{1}H NMR and ^{13}C NMR spectra of pyrroles **1a**, **9**, **10**, **15-18**, and **2a**	S2-S17
2. Single-crystal XRD of pyrrole 2a	S18-S19

1.1. H NMR and ^{13}C NMR spectra

1-(4-Fluorophenyl)-2-methyl-5-(4-(methylsulfonyl)phenyl)-1*H*-pyrrole-3-carbonitrile **1a**

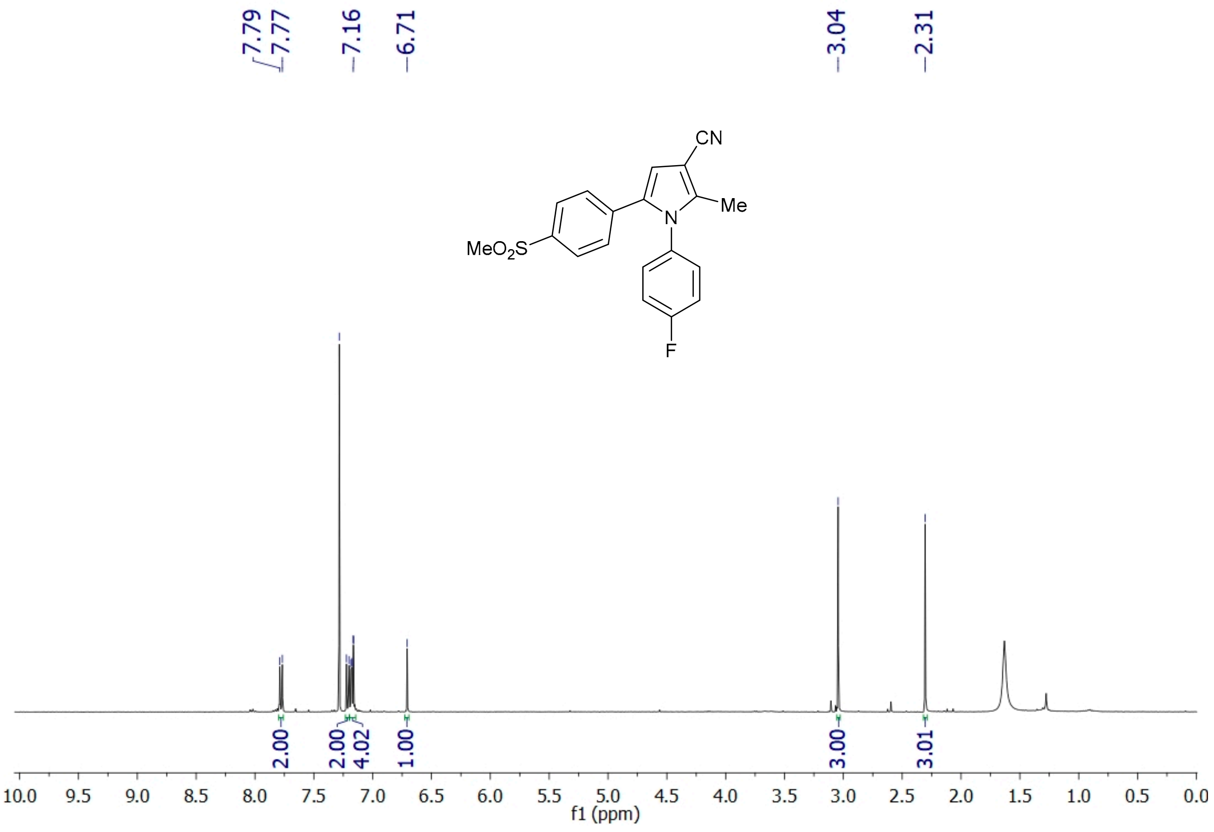

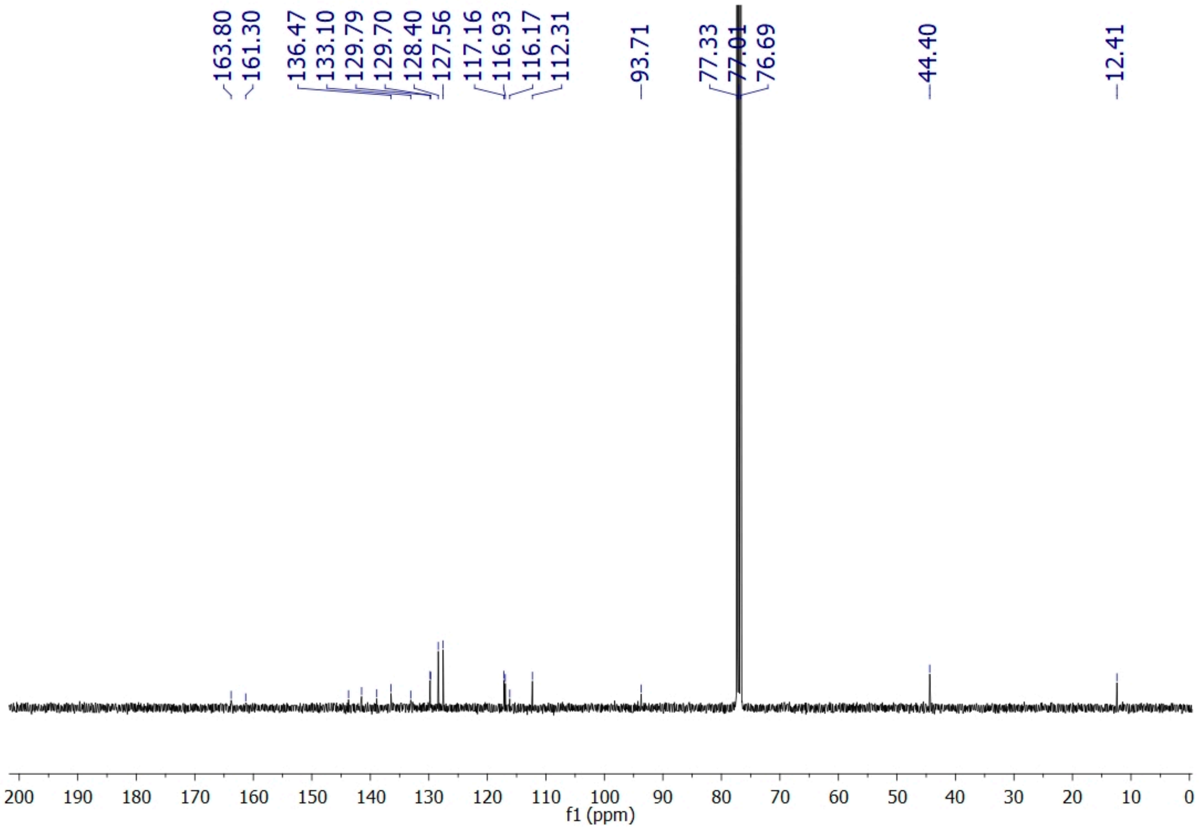
163.80
161.30
136.47
133.10
129.79
129.70
128.40
127.56
117.16
116.93
116.17
112.31
93.71
77.33
77.01
76.69
44.40
12.41
200 190 180 170 160 150 140 130 120 110 100 90 80 70 60 50 40 30 20 10 0
f1 (ppm)

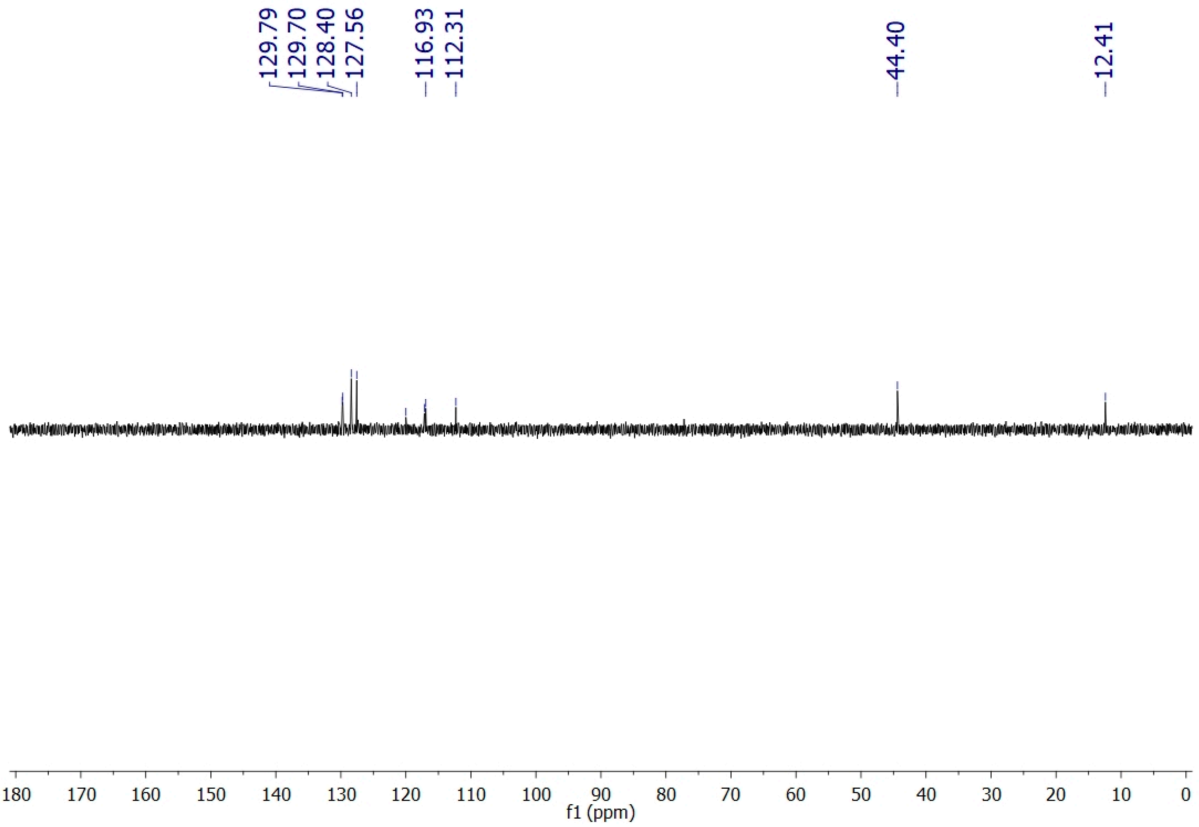
129.79
129.70
128.40
127.56
116.93
112.31
44.40
12.41
180 170 160 150 140 130 120 110 100 90 80 70 60 50 40 30 20 10 0
f1 (ppm)

2-Methyl-5-(4-(methylsulfonyl)phenyl)-1-phenethyl-1*H*-pyrrole-3-carbonitrile **9**

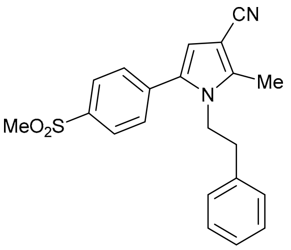

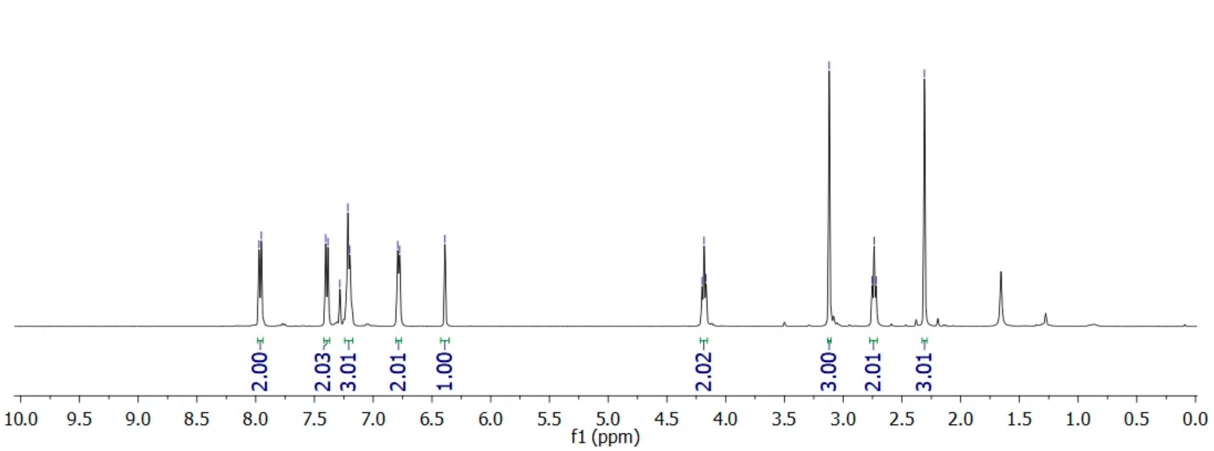

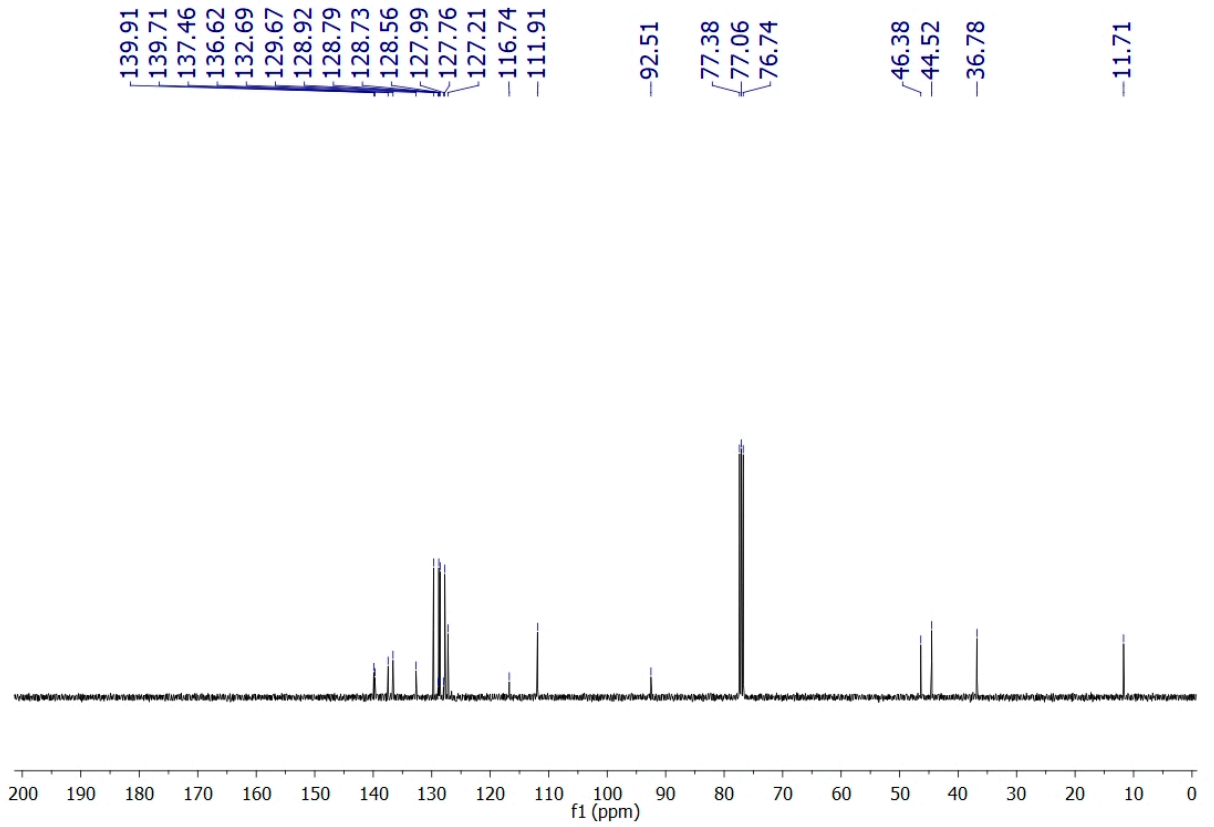

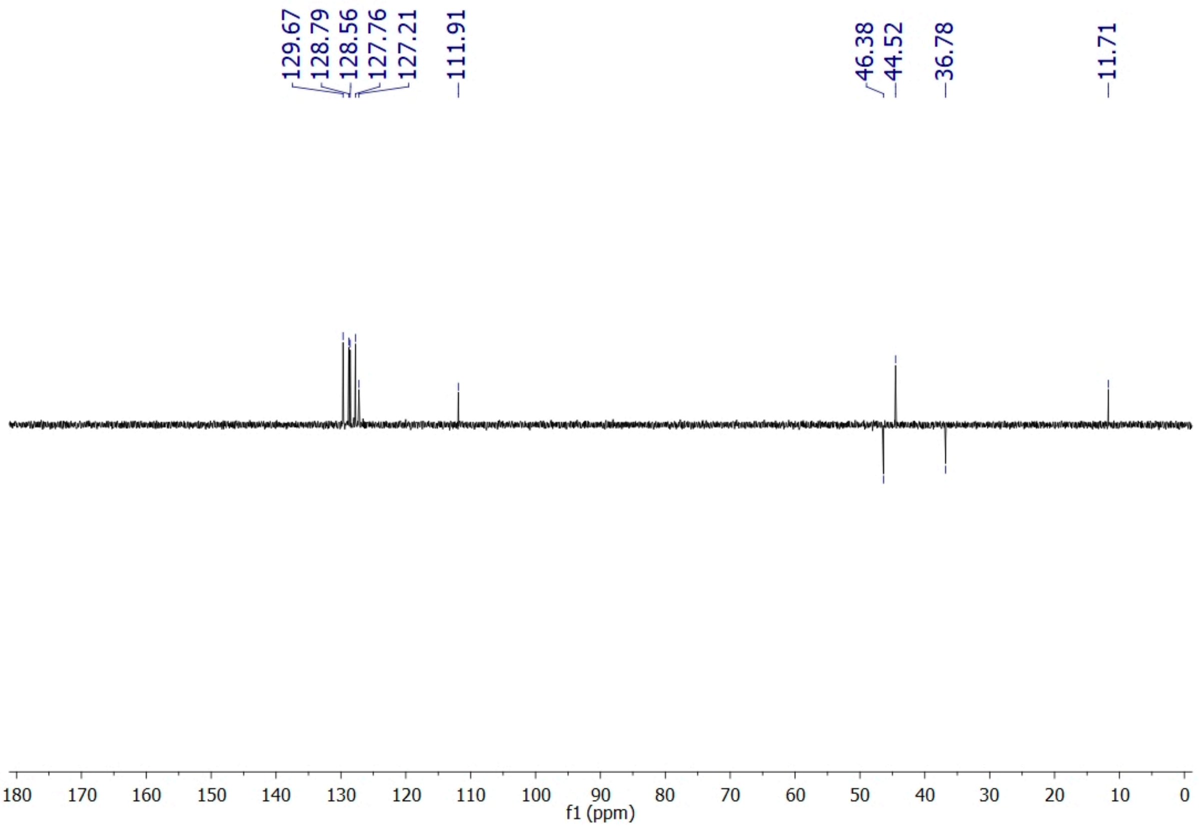
129.67
128.79
128.56
127.76
127.21
111.91
46.38
44.52
36.78
11.71
180 170 160 150 140 130 120 110 100 90 80 70 60 50 40 30 20 10 0
f1 (ppm)

1-Benzyl-5-(4-(methylsulfonyl)phenyl)-2-phenyl-1*H*-pyrrole-3-carbonitrile **10**

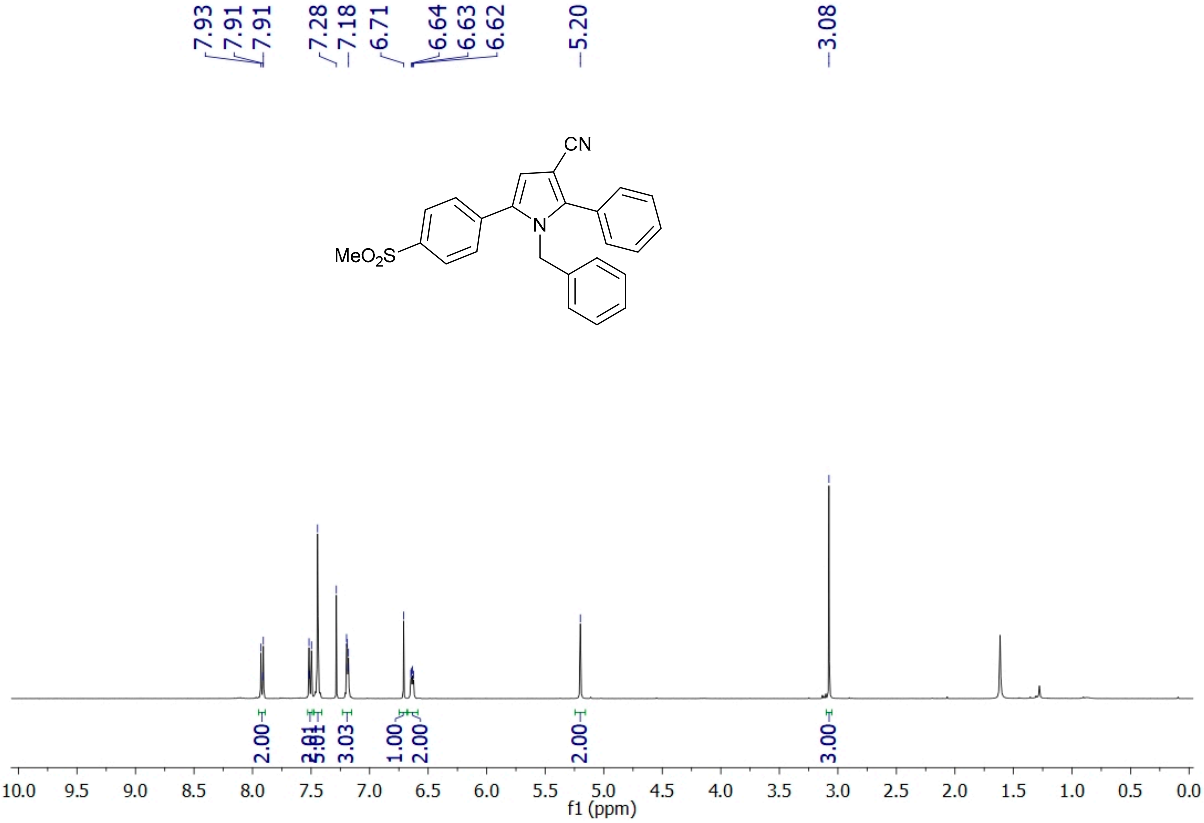

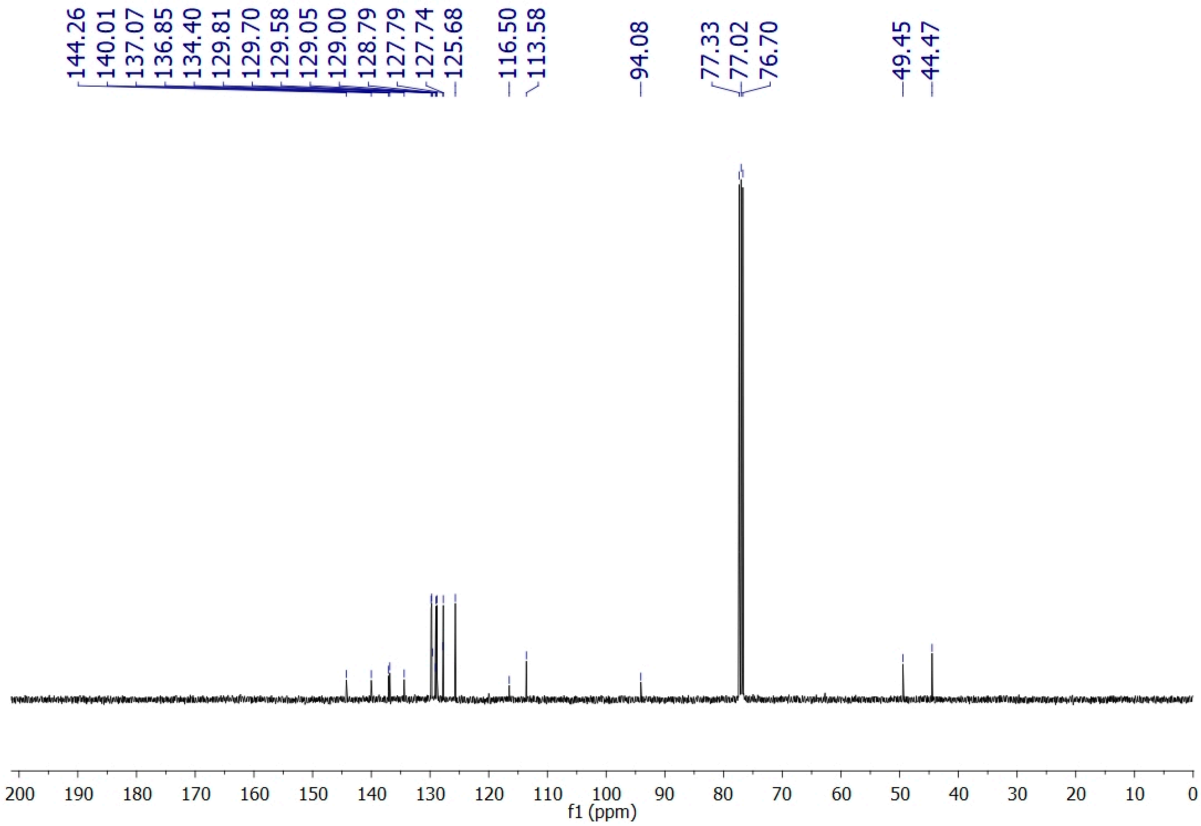

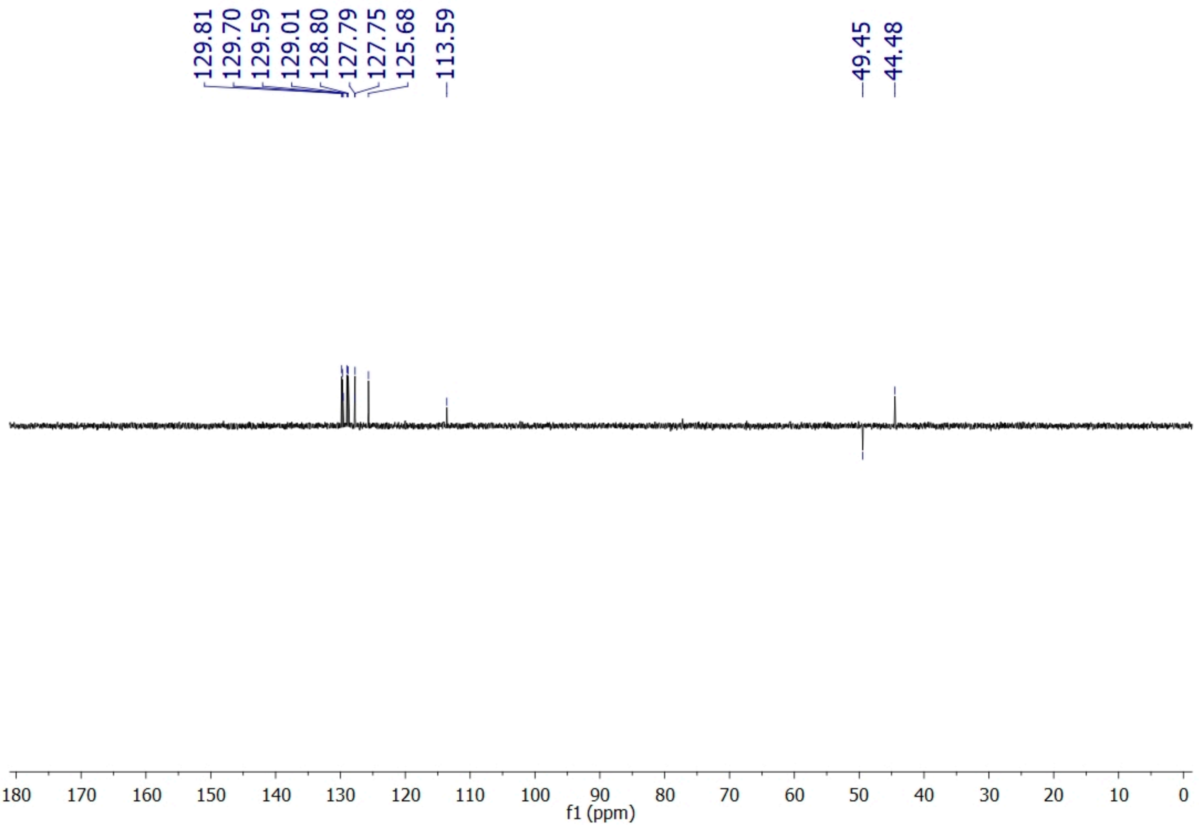
129.81
129.70
129.59
129.01
128.80
127.79
127.75
125.68
113.59
49.45
44.48
180 170 160 150 140 130 120 110 100 90 80 70 60 50 40 30 20 10 0
f1 (ppm)

1,5-Bis(4-chlorophenyl)-2-methyl-1*H*-pyrrole-3-carbonitrile **15**

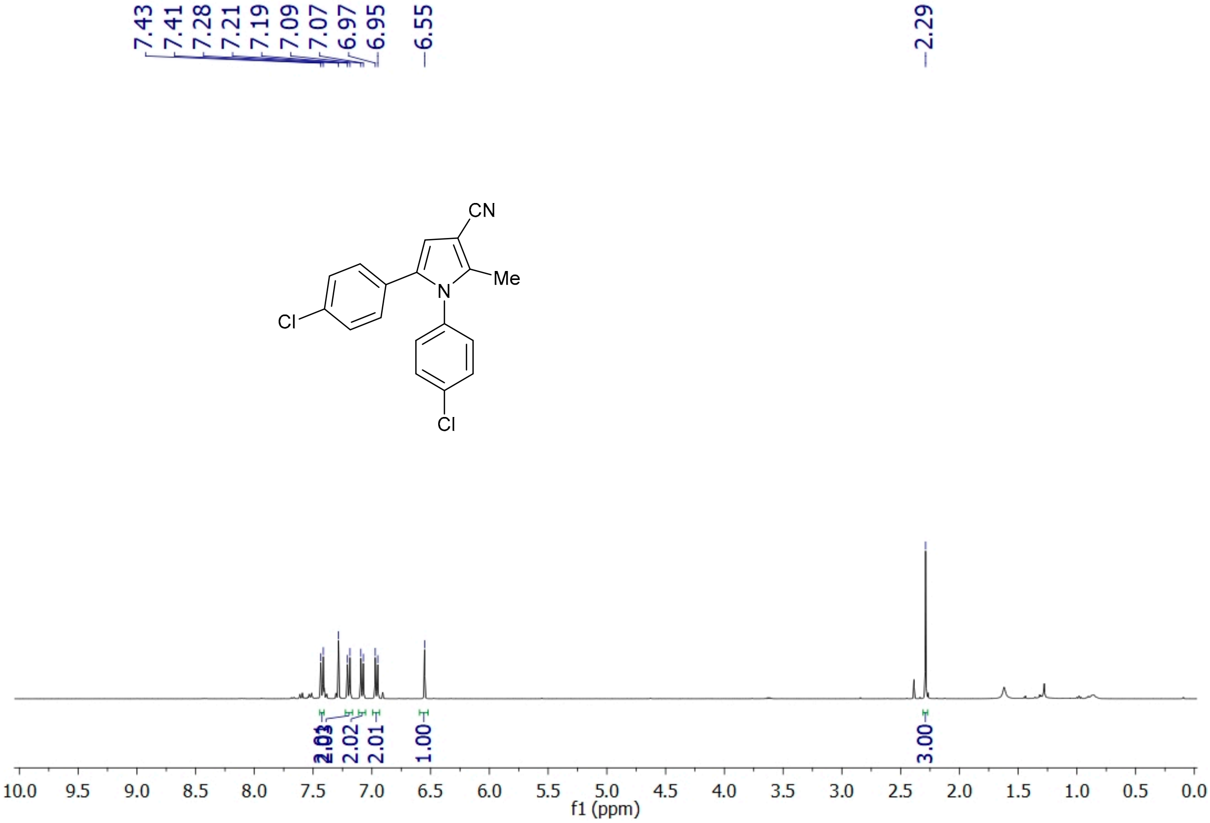

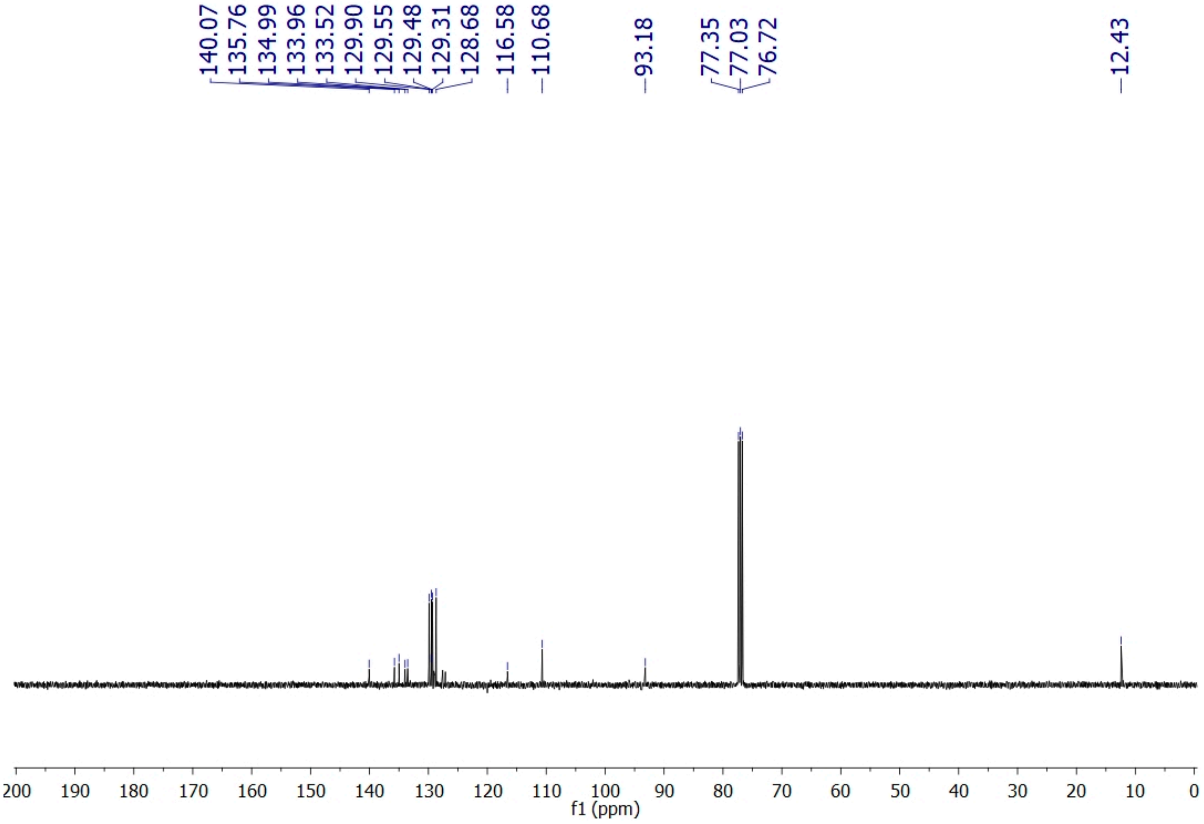

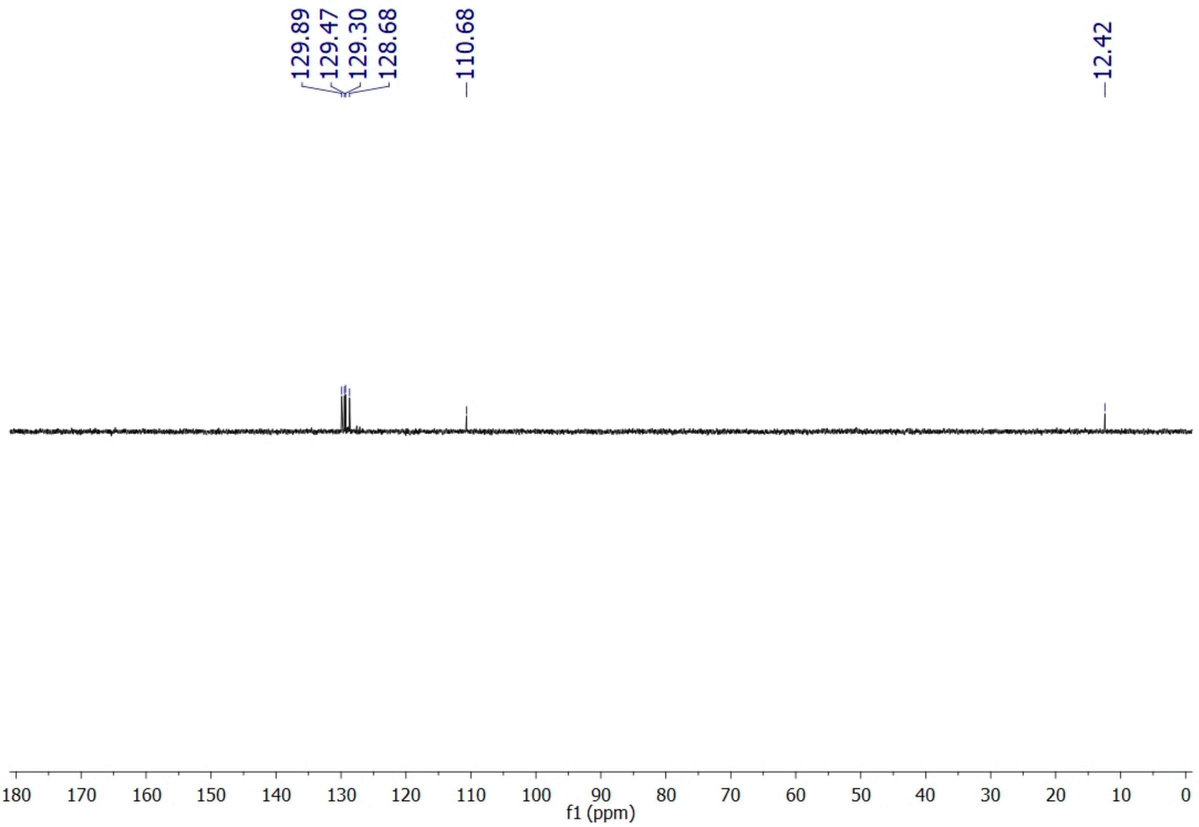

1-(4-Fluorophenyl)-2-methyl-5-(p-tolyl)-1*H*-pyrrole-3-carbonitrile **16**

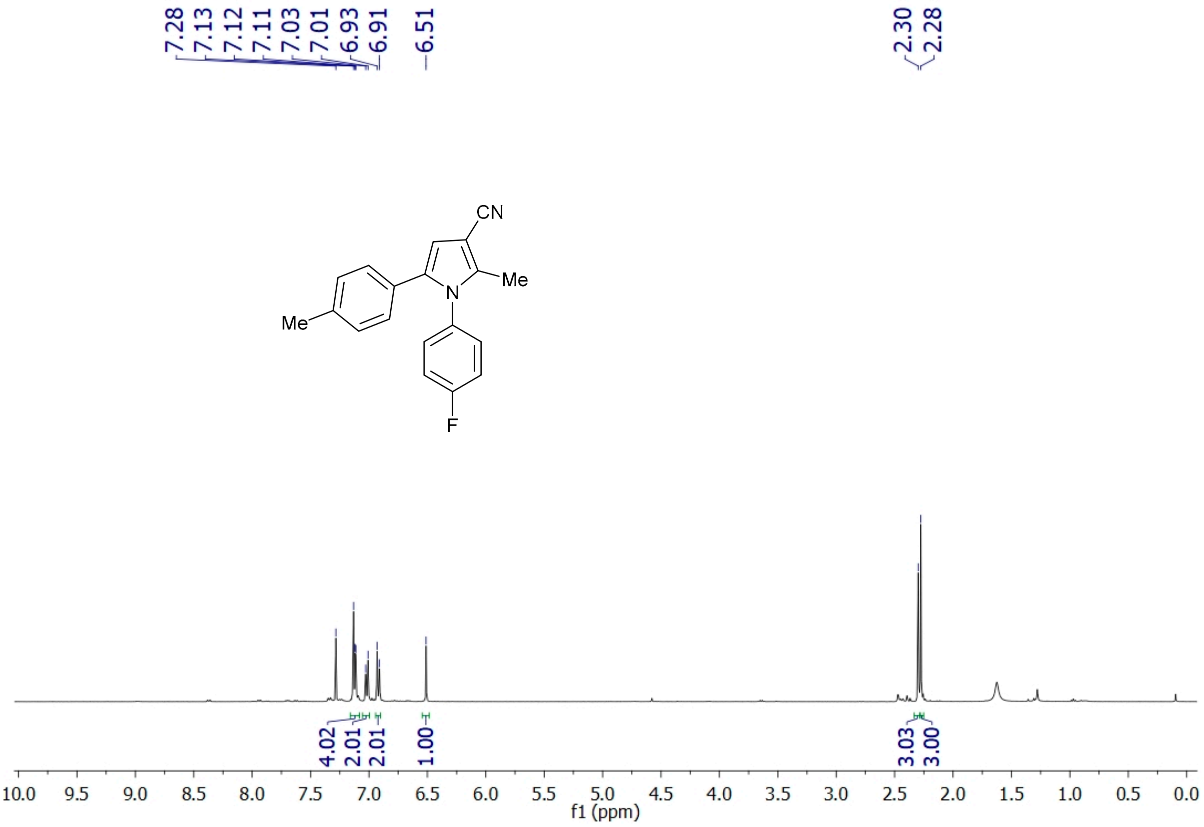

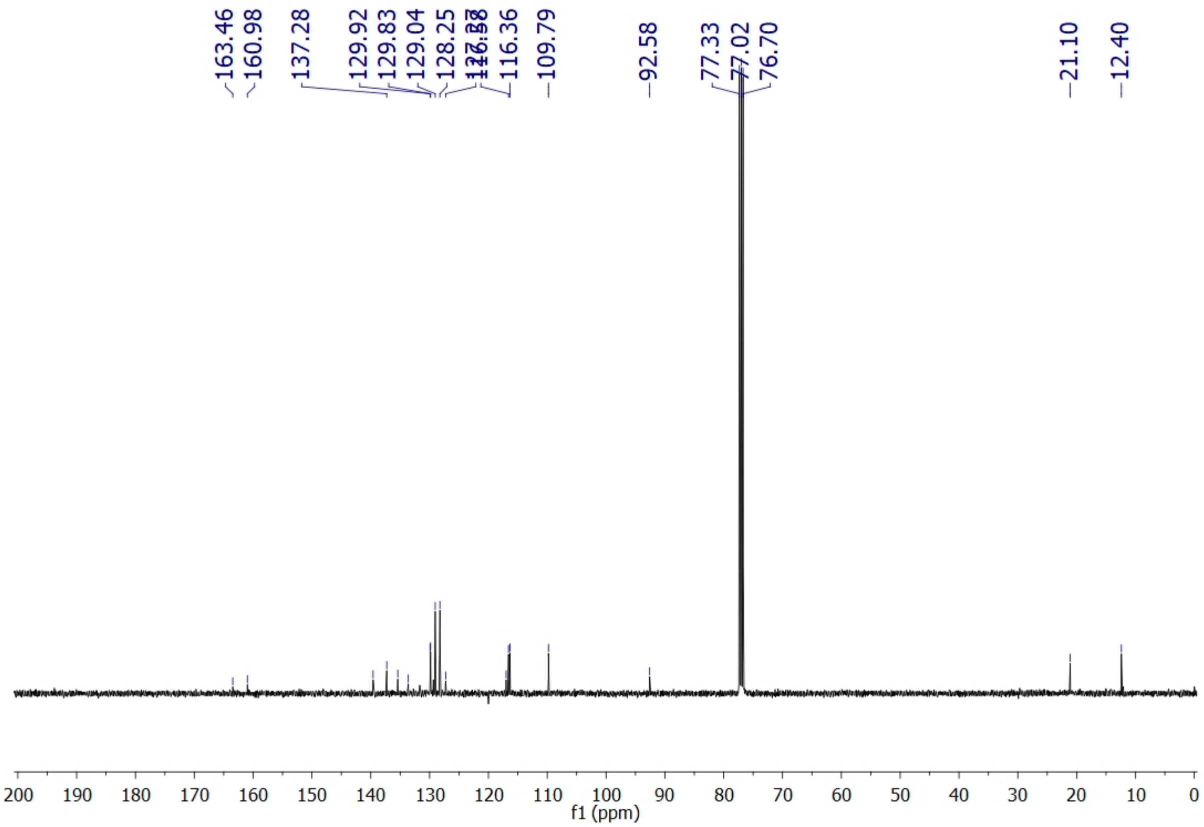
163.46
160.98
137.28
129.92
129.83
129.04
128.25
116.36
109.79
92.58
77.33
77.02
76.70
21.10
12.40
200 190 180 170 160 150 140 130 120 110 100 90 80 70 60 50 40 30 20 10 0
f1 (ppm)

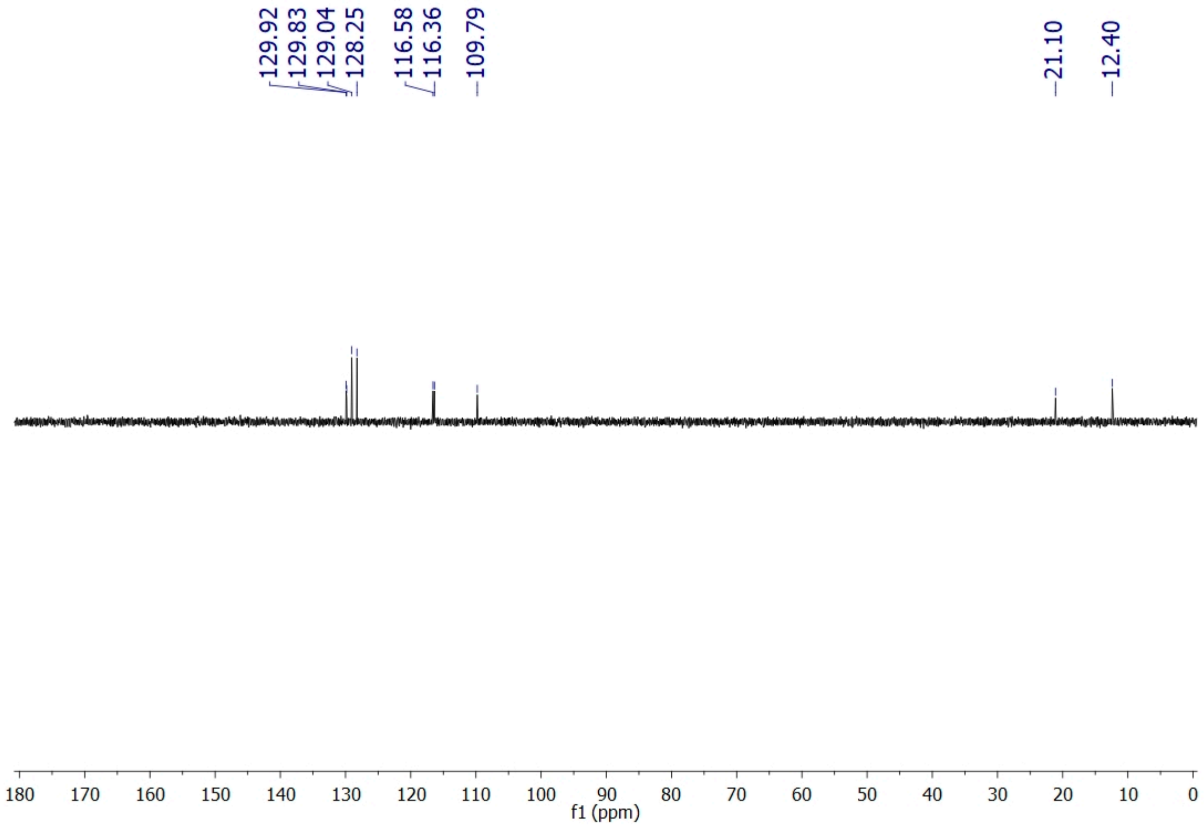
129.92
129.83
129.04
128.25
116.58
116.36
109.79
21.10
12.40
180 170 160 150 140 130 120 110 100 90 80 70 60 50 40 30 20 10 0
f1 (ppm)

1-(4-Fluorophenyl)-5-(4-methoxyphenyl)-2-methyl-1*H*-pyrrole-3-carbonitrile **17**

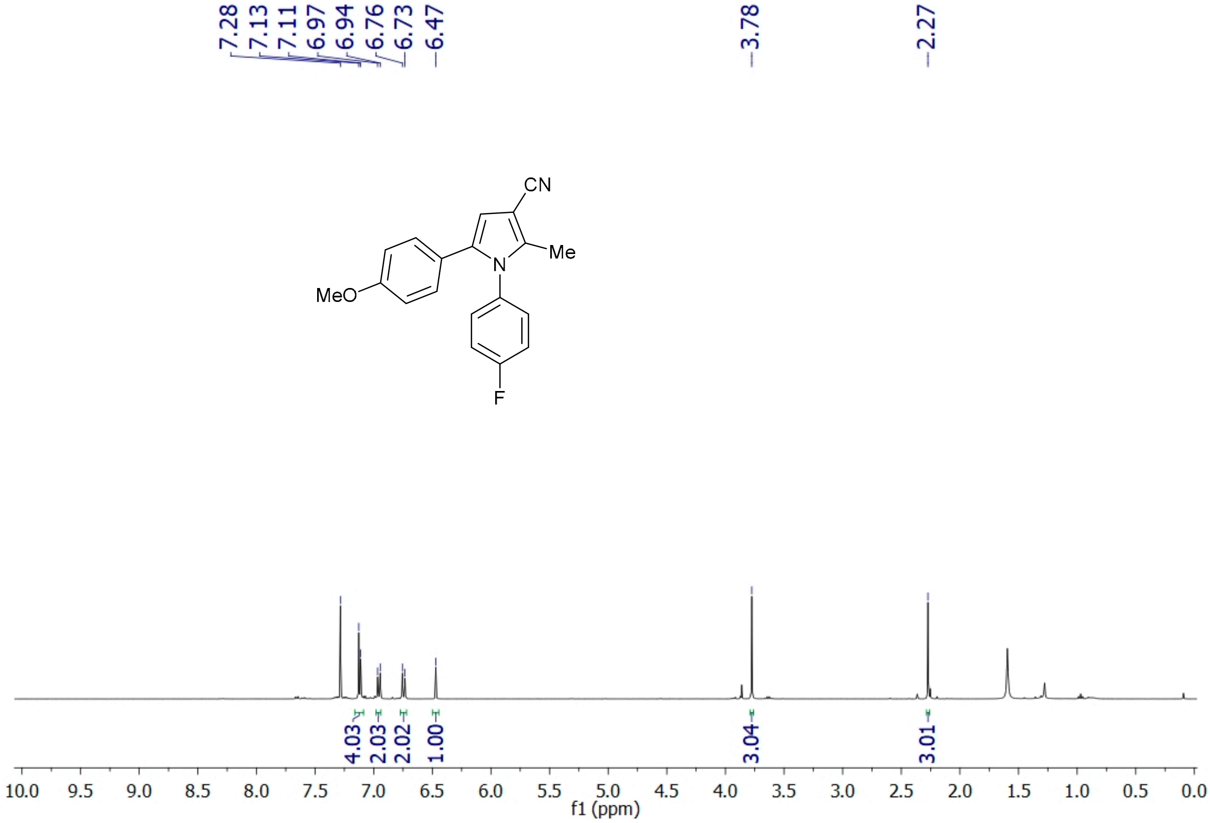

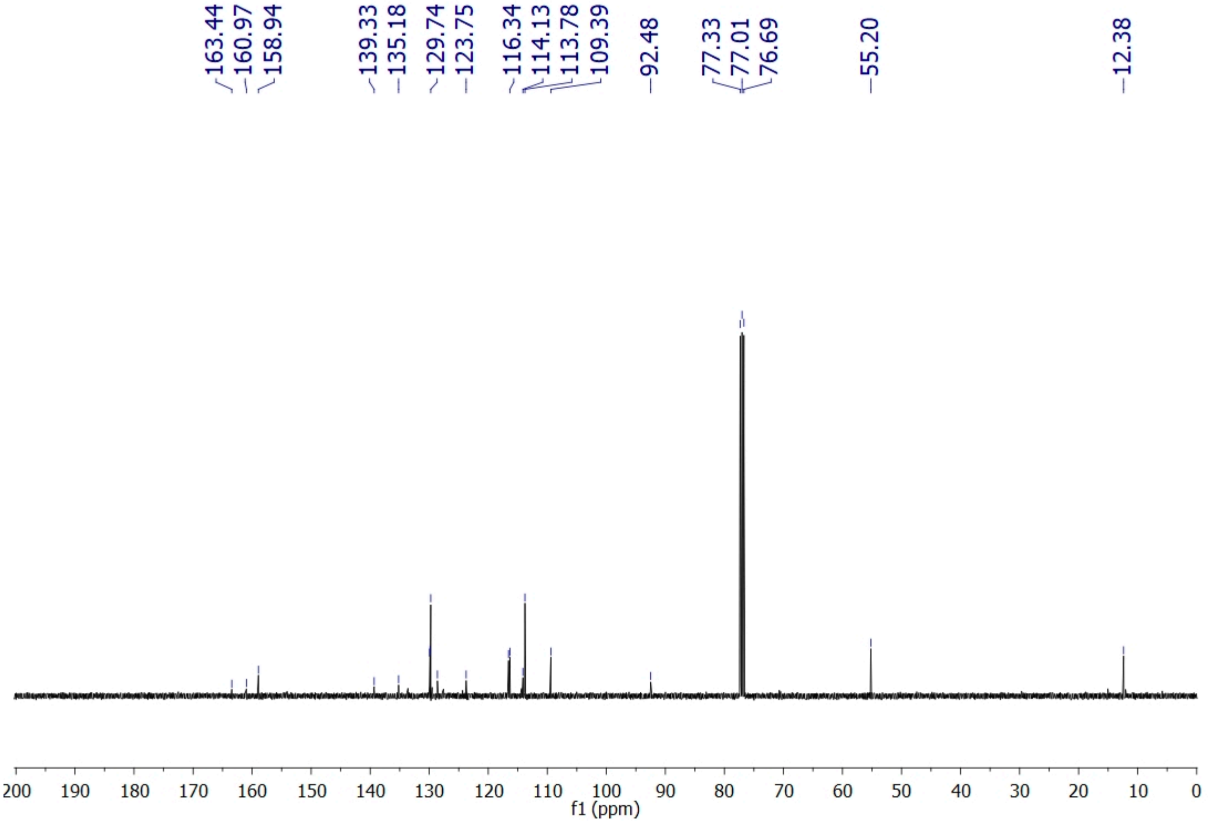

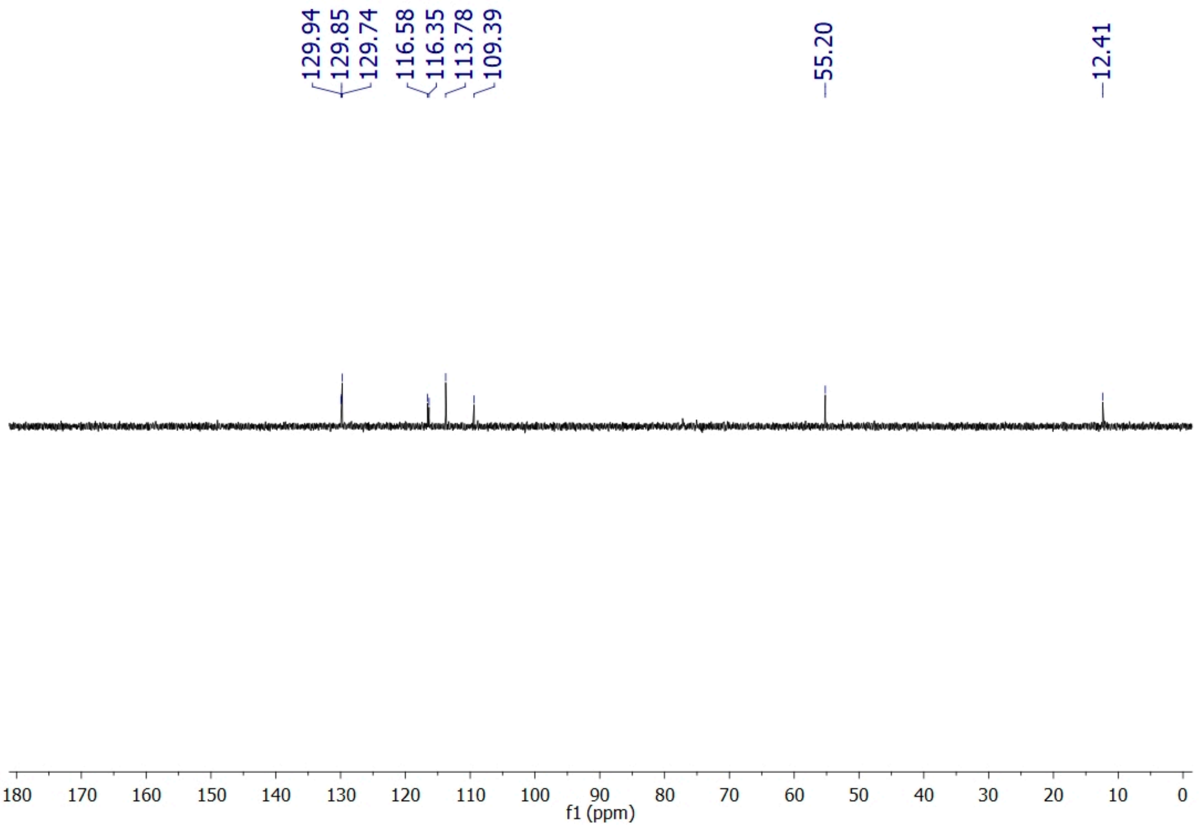

1,5-Bis(4-chlorophenyl)-2-methyl-1H-pyrrole-3-carbaldehyde **18**

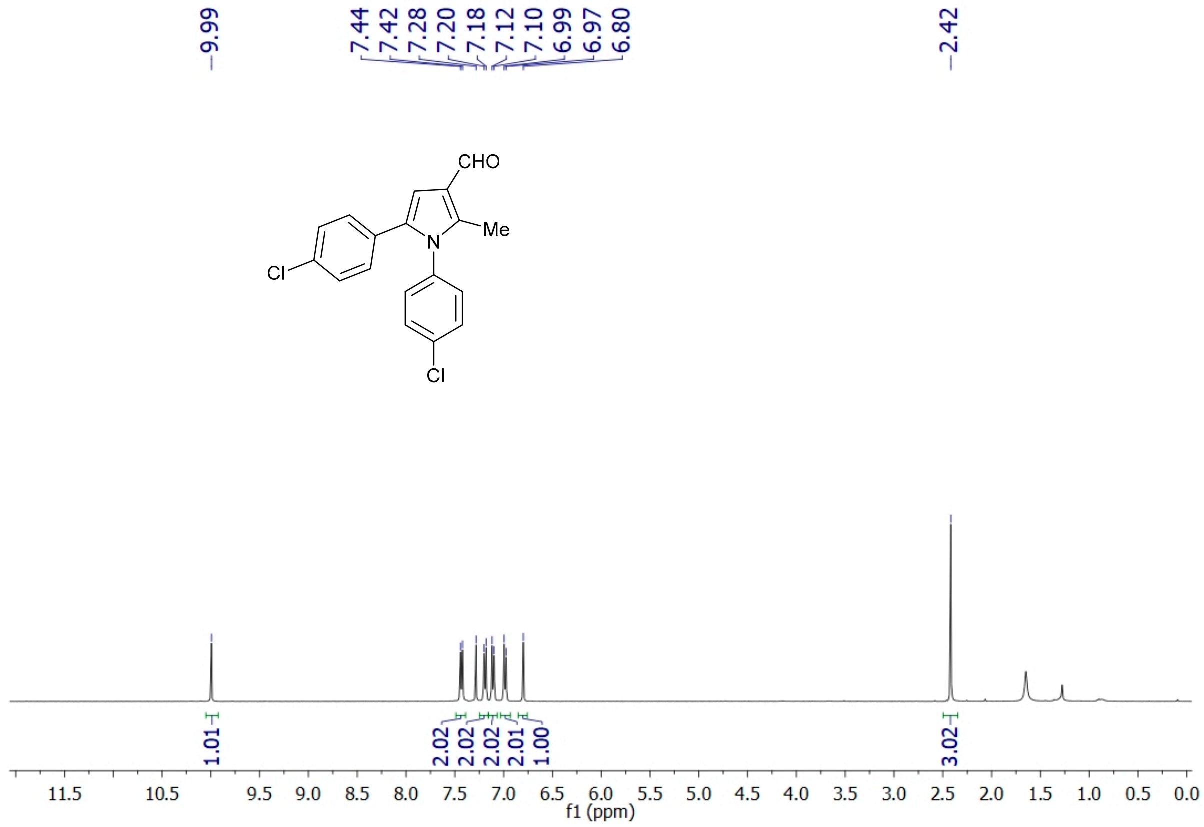

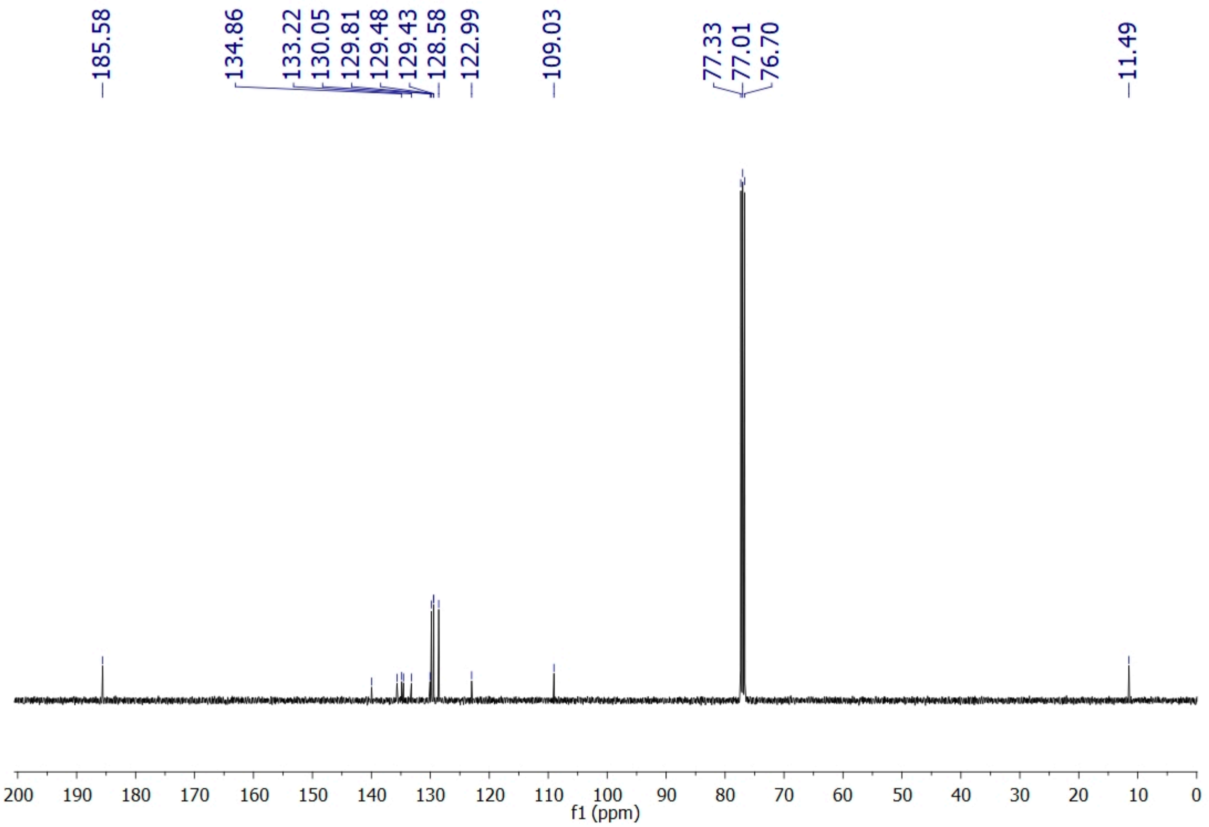
185.58
134.86
133.22
130.05
129.81
129.48
129.43
128.58
122.99
109.03
77.33
77.01
76.70
11.49
200 190 180 170 160 150 140 130 120 110 100 90 80 70 60 50 40 30 20 10 0
f1 (ppm)

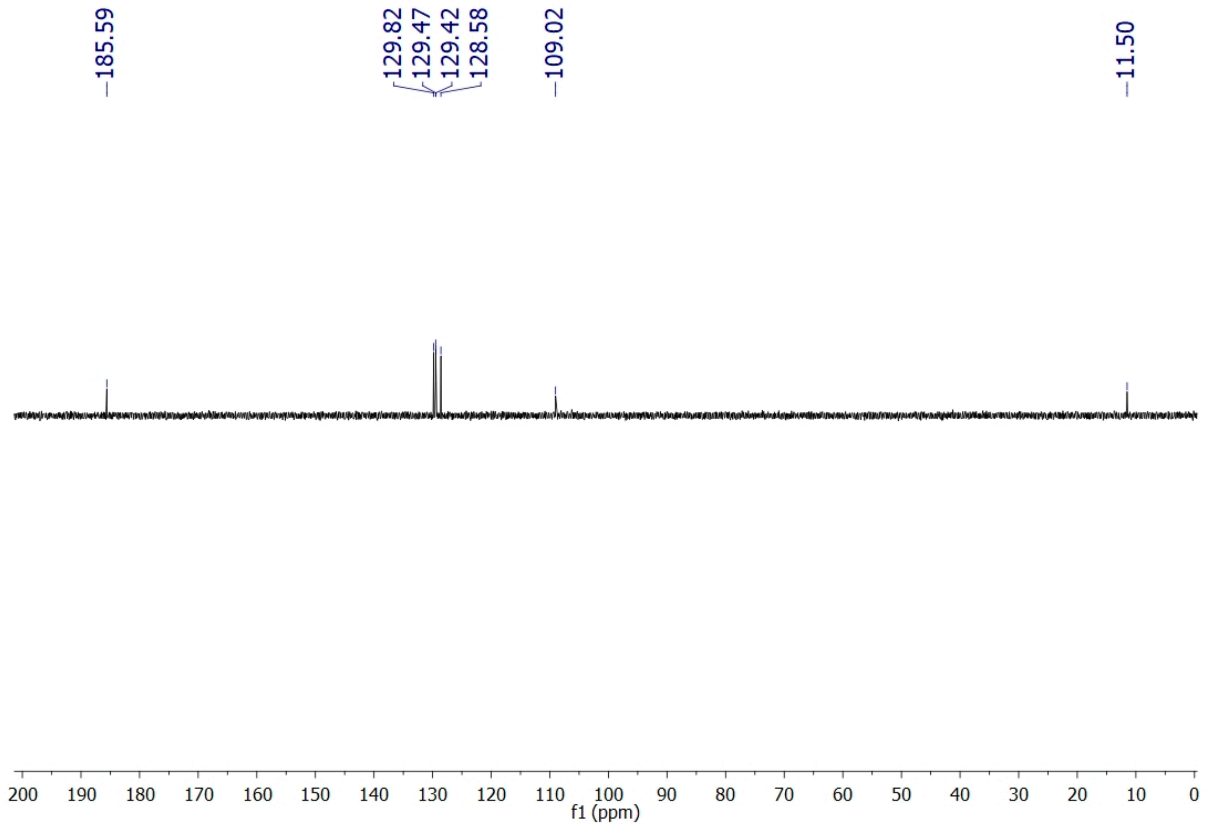
185.59
129.82
129.47
129.42
128.58
109.02
11.50
200 190 180 170 160 150 140 130 120 110 100 90 80 70 60 50 40 30 20 10 0
f1 (ppm)

1-((1,5-Bis(4-chlorophenyl)-2-methyl-1*H*-pyrrol-3-yl)methyl)-4-methylpiperazine **2a**

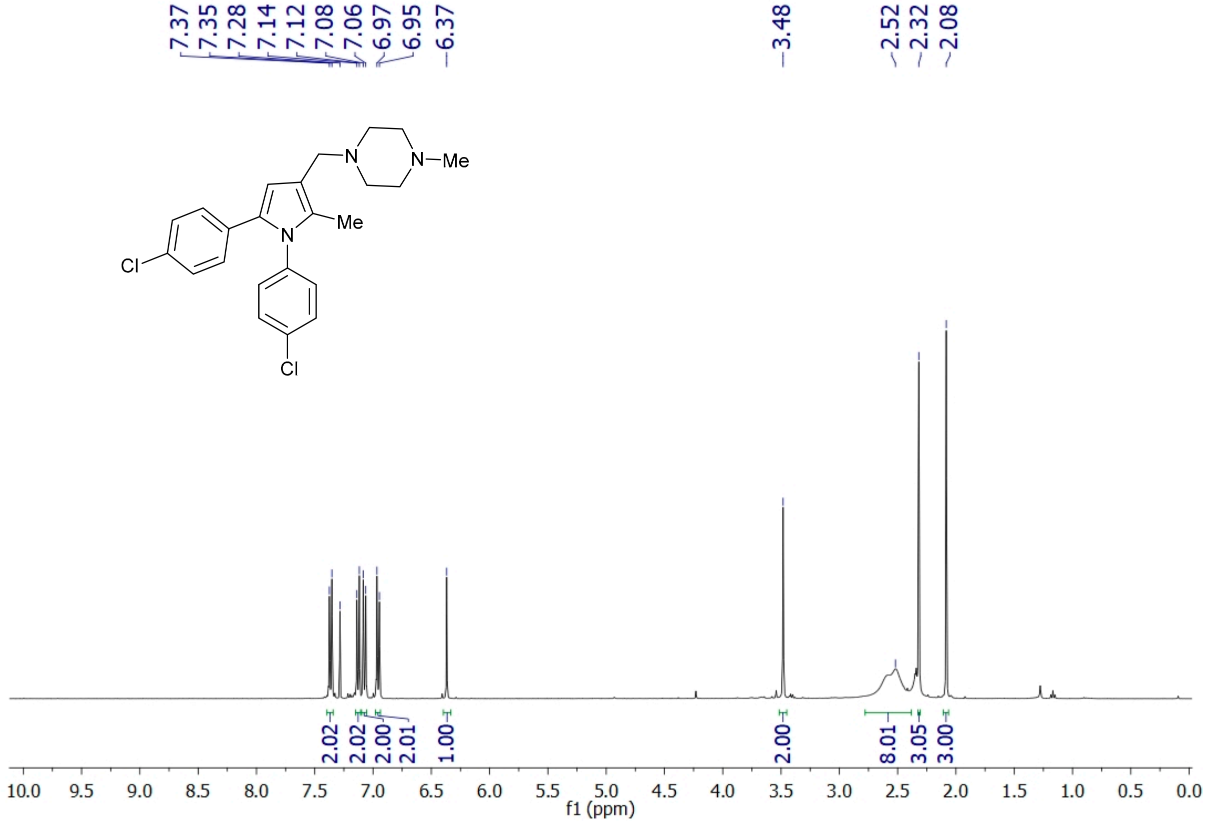

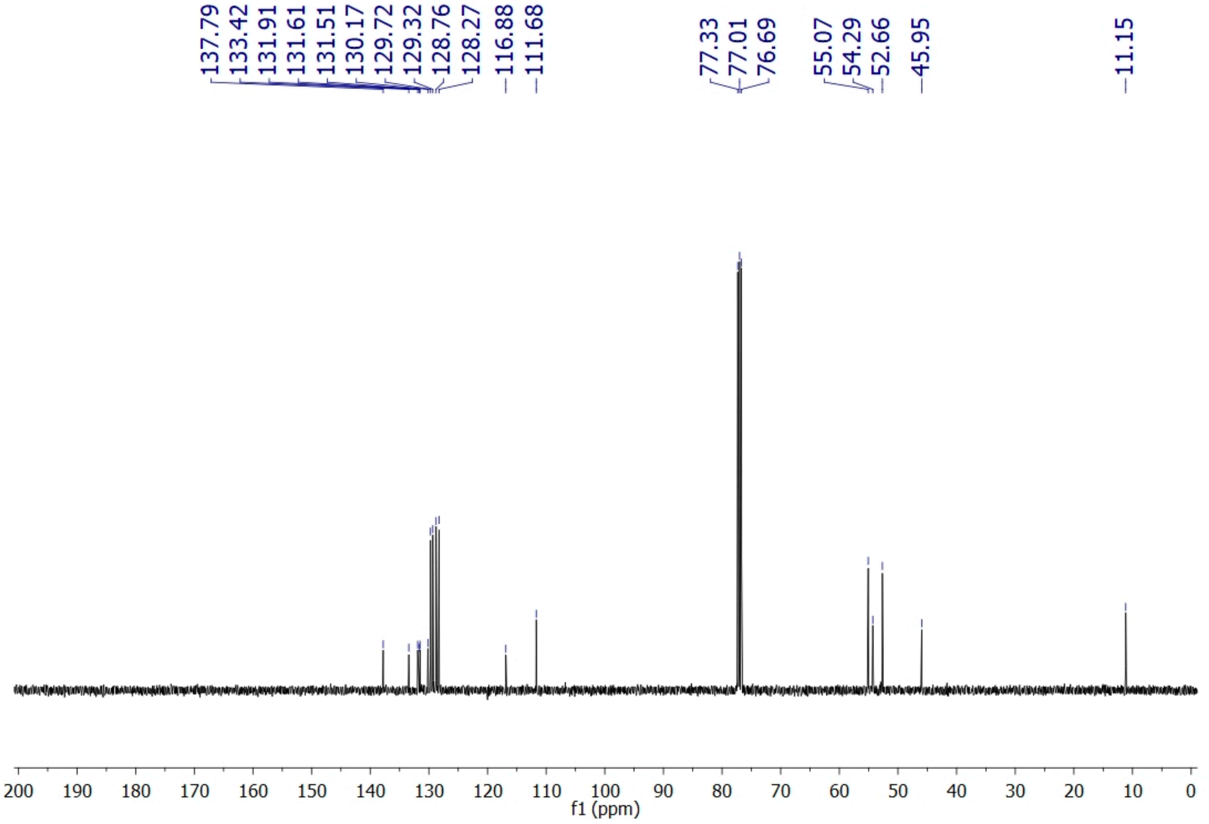

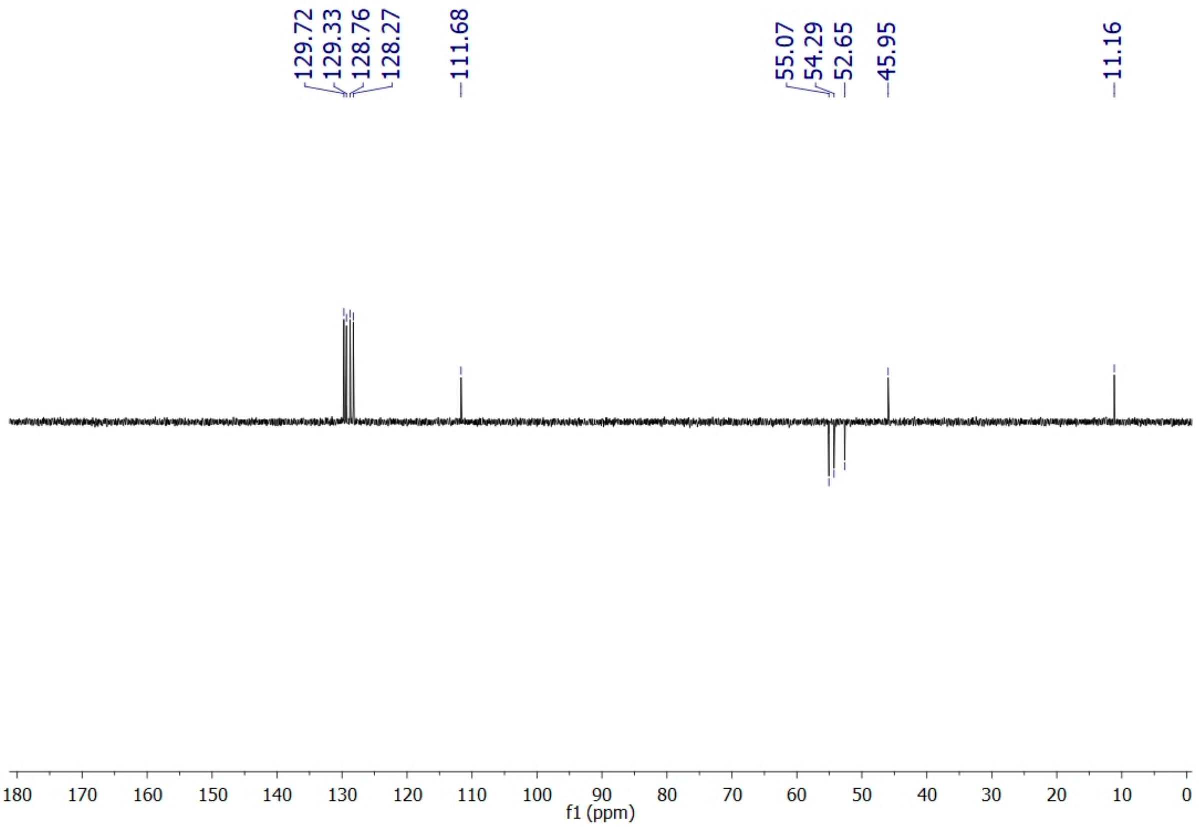

2. Single-crystal XRD of BM212 (product 2a)

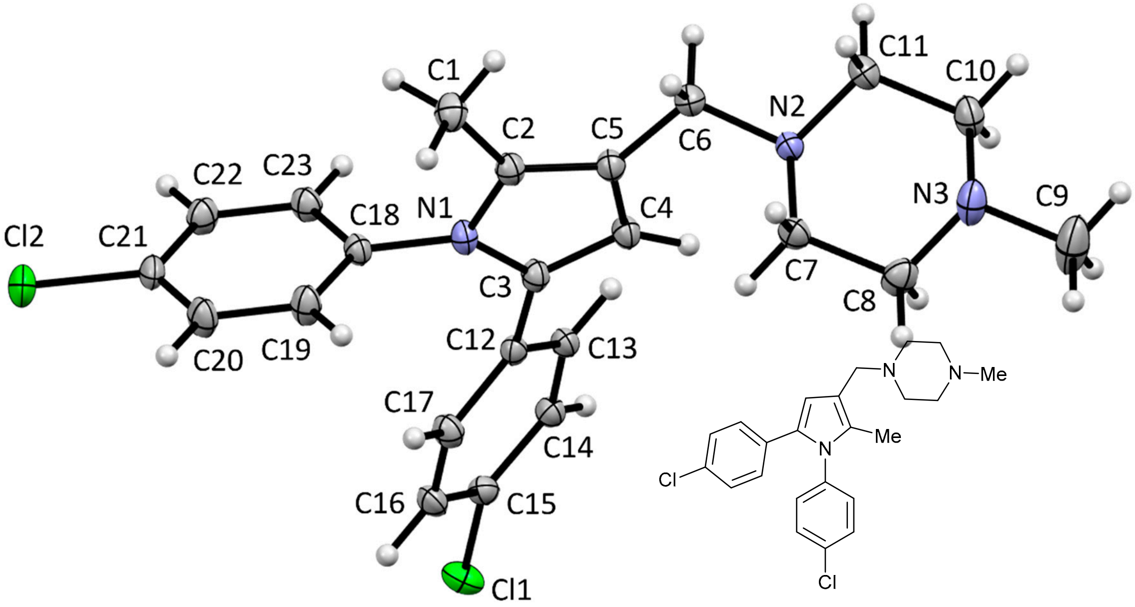

Figure S1. X-ray single crystal structure of BM212 **2a** (CCDC 2191163) (displacement ellipsoids are drawn at the 50% probability level).

Table S1. Crystal data and structure refinement for product **2a** (CCDC 2191163).

Empirical formula	$C_{23}H_{25}Cl_2N_3$
Formula weight	414.36
Temperature	100(2) K
Crystal system	Triclinic
Space group	P-1
Unit cell dimensions	a = 9.1454(10) Å a= 100.119(4)°.<br>b = 11.1138(12) Å b= 100.255(4)°.<br>c = 11.2090(12) Å g = 106.088(4)°.
Volume	1046.2(5) Å^3
Z	2
Density (calculated)	1.315 Mg/m^3
F(000)	436
Reflections collected	26513
Independent reflections	8999 [R(int) = 0.0570]
Max. and min. transmission	0.9150 and 0.9030
Goodness-of-fit on F^2	1.025
Final R indices [I>2sigma(I)]	R1 = 0.0533, wR2 = 0.1200
R indices (all data)	R1 = 0.0892, wR2 = 0.1380
